# Fatigue-free artificial ionic skin toughened by self-healable elastic nanomesh

**DOI:** 10.1038/s41467-022-32140-3

**Published:** 2022-07-29

**Authors:** Jiqiang Wang, Baohu Wu, Peng Wei, Shengtong Sun, Peiyi Wu

**Affiliations:** 1 State Key Laboratory for Modification of Chemical Fibers and Polymer Materials, College of Chemistry, Chemical Engineering and Biotechnology & Center for Advanced Low-dimension Materials, Donghua University, 2999 North Renmin Road, Shanghai, 201620 China; 2 Jülich Centre for Neutron Science (JCNS) at Heinz Maier-Leibnitz Zentrum (MLZ) Forschungszentrum Jülich, Lichtenbergstr. 1, 85748 Garching, Germany

**Keywords:** Gels and hydrogels, Sensors and biosensors, Sensors and probes, Biomaterials

## Abstract

Robust ionic sensing materials that are both fatigue-resistant and self-healable like human skin are essential for soft electronics and robotics with extended service life. However, most existing self-healable artificial ionic skins produced on the basis of network reconfiguration suffer from a low fatigue threshold due to the easy fracture of low-energy amorphous polymer chains with susceptible crack propagation. Here we engineer a fatigue-free yet fully healable hybrid ionic skin toughened by a high-energy, self-healable elastic nanomesh, resembling the repairable nanofibrous interwoven structure of human skin. Such a design affords a superhigh fatigue threshold of 2950 J m^−2^ while maintaining skin-like compliance, stretchability, and strain-adaptive stiffening response. Moreover, nanofiber tension-induced moisture breathing of ionic matrix leads to a record-high strain-sensing gauge factor of 66.8, far exceeding previous intrinsically stretchable ionic conductors. This concept creates opportunities for designing durable ion-conducting materials that replicate the unparalleled combinatory properties of natural skins more precisely.

## Abstract (web summary)

Developing robust skin-like sensing materials is essential for soft electronics and robotics with extended service life. Here, inspired by the repairable nanofibrous structure of human skin, the authors engineer a fatigue-resistant artificial ionic skin toughened by self-healable elastic nanomesh.

## Introduction

Developing skin-like robust sensing materials is highly desirable for future human-machine interfaces and soft robotics but difficult to realize, in particular for the combinatory properties that generally require mutually exclusive material designs involving conductivity, stretchability, softness, toughness, healability, and durability^1–4^. In terms of the critical requirements for long service life, the material should not only heal itself upon damage but also resist crack propagation during fatigue loads. However, striking a balance between self-healability and fatigue resistance has proved to be a challenging task for stretchable ionic conductors (also known as ionic skins)-the most important artificial analogs of human skin with similar moduli and ion-conducting nature^5–7^. Noteworthily, most self-healable ionic skins are produced by incorporating dynamic covalent or physical crosslinks in the ion-conducting network which reconfigures through chain re-arrangement^8–10^. Such an interchain dynamic design does not essentially contribute to fatigue-fracture resistance, since the low energy required to fracture a single layer of amorphous polymer chains determines susceptible crack propagation upon repeatable loads^11^. Consequently, self-healing ionic skins even toughened by sacrificial or energy-dissipating components may resist crack propagation under monotonic load, but still, easily fail in cyclic loads which are unaffected by the complementary dissipation mechanisms^12,13^. Recent studies show that an elastomer’s fatigue threshold (the minimal fracture energy below which cracks cease to propagate under cyclic loading) may be largely enhanced by embedding high-energy hard domains in the matrix, such as nanocrystallites^12,14^, aligned lamellar/fibrillar structures^11,15–17^, and high-contrast segregated nanophases^18–20^. Yet, the incorporated hard domains are usually irreparable, and the only reported anti-fatigue polyampholyte hydrogels with healable hard phases exhibit a limited fatigue threshold (*Γ*_0_) of ∼150 J m^−2^ ^19^. Alternatively, introducing swollen covalent-network microspheres for reversible entanglements with a linear network has been recently reported to also enable high fatigue resistance and self-healability, but the resulting mechanical strength is relatively low due to the intrinsic softness of embedded microdomains^21^.

**Fig. 1:**
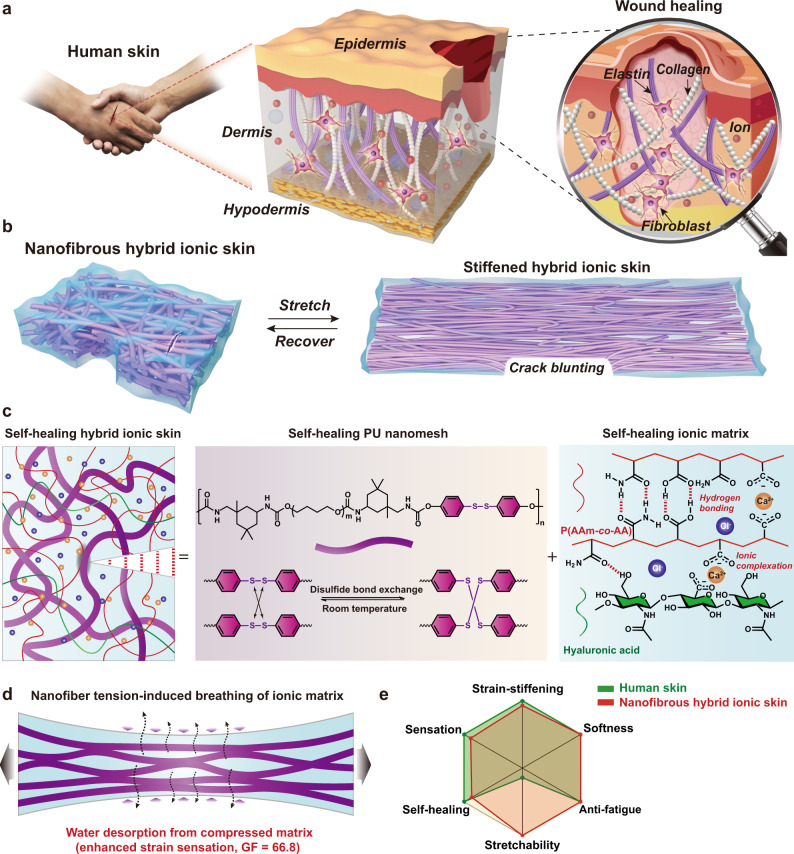
Design of fatigue-free and self-healable nanofibrous hybrid ionic skin. **a** Schematic ion-rich, nanofibrous composite structure and wound-repairing mechanism of human skin. Both the collagen fibrils and elastin matrix are healable with the aid of fibroblasts. **b** Schematic structural changes of self-healable nanofibrous hybrid ionic skin upon reversible stretching. Hybrid ionic skin rapidly stiffens as deformed and is highly anti-fatigue-fracture through a nanofiber-induced crack blunting mechanism. **c** Schematic illustrations of the hybrid structure and respective self-healing mechanisms of PU nanomesh scaffold and ionic matrix. **d** Nanofiber tension forces the hygroscopic ionic matrix to reversibly breathe moisture in air, leading to substantially enhanced strain sensation (gauge factor, GF = 66.8). **e** A rough comparison of the sensing and mechanical performance between human skin and nanofibrous hybrid ionic skin.

In sharp contrast, human skin well reconciles this trade-off between healability and fatigue resistance, originating from its ion-rich and hierarchically arranged nanofibrous yet repairable structure, defined by a stiff collagen fibril scaffold embedded in the soft interwoven elastin matrix (Fig. 1a). These two phases not only heal with the aid of dermal fibroblasts upon wound but also impart very high fracture toughness to human skin by pinning crack tip at the hard collagen nanofibrils. Therefore, human skin can sustain tear fractures as well as repeatable deformations like muscles (*Γ*_0_ ∼ 1000 J m^−2^) over one million cycles per year without fatigue^22,23^. Moreover, the nanofibrous structure makes human skin both soft and firm, i.e., the soft elastin network ensures structural compliance and stiff collagen fibrils resist large deformations that may threaten tissue integrity, resulting in unique exponential stiffening with J-shaped stress-strain behavior^24^.

Inspired by skin’s repairable nanofibrous structure, here we design an artificial sensing ionic skin by embedding a high-energy self-healable elastic nanomesh scaffold into another self-healable soft ionic matrix (Fig. 1b, c). Such a hybrid design leads to superhigh fracture energy (16.3 kJ m^−2^) and fatigue threshold (2950 J m^−2^) while maintaining skin-like self-healability, softness (modulus ∼1.8 MPa), stretchability (680%), and strain-stiffening response (stiffened to 67.5 MPa, corresponding to 37 times stiffness enhancement). Moreover, the ionic conductivity of the ionic matrix is highly sensitive to humidity (RH 20%: 5.5 × 10^−4^ S m^−1^; RH 90%: 4.8 S m^−1^). The tension-induced alignment of nanofibers forces the hygroscopic ionic matrix to reversibly breathe moisture in the air (Fig. 1d), resulting in substantially enhanced tensile sensation with a record-high gauge factor of 66.8 for intrinsically stretchable ionic conductors. We thus report an artificial ionic skin that resembles or even surpasses human skin in a few highlighted sensing/mechanical properties (sensation, softness, stretchability, self-healing, strain-stiffening, anti-fatigue) (Fig. 1e and Supplementary Table 1). Besides, the hybrid ionic skin is transparent, anti-freezing, ambient-stable, and adhesive, further endowing it with great potential to be applied in various sensing scenarios.

## Results

### Design of nanofibrous hybrid ionic skin

**Fig. 2:**
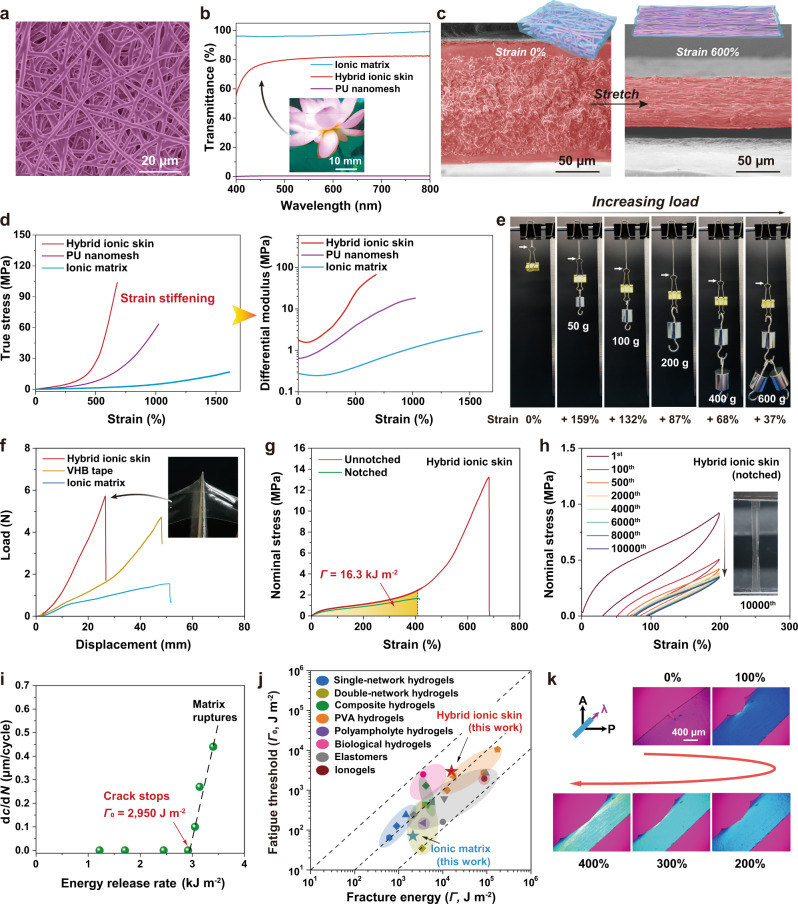
Morphology and mechanical properties of hybrid ionic skin. **a** SEM image of self-healing PU nanomesh. **b** Transmittance of hybrid ionic skin, PU nanomesh, and ionic matrix. **c** Cross-sectional SEM images of hybrid ionic skin before and after stretching. **d** True stress-strain and corresponding differential modulus curves. **e** Strain-stiffening demonstration of a hybrid ionic skin strip (15 × 3 × 0.13 mm^3^) with increasing loads. **f** Puncture force-displacement curves of hybrid ionic skin (thickness ~130 μm), ionic matrix (1 mm), and commercial VHB (Very High Bond) tape (0.5 mm). **g** Nominal stress-strain curves of unnotched and notched hybrid ionic skins. **h** Cyclic tensile curves of notched hybrid ionic skin at 200% strain (inset: stretched sample at the 10,000th cycle). **i** Crack extension per loading cycle, d*c*/d*N*, versus energy release rate for hybrid ionic skin. **j** Comparison of the fatigue threshold (*Γ*_0_) and fracture energy (*Γ*) of hybrid ionic skin with previously reported anti-fatigue hydrogels and elastomers. **k** POM images of a notched hybrid ionic skin as gradually stretched to 400% strain. All the images were taken in the presence of 530 nm tint plate at the azimuth angle of 45^o^. Source data are provided as a Source Data file.

Transparent nanofibrous hybrid ionic skin was delicately designed by combining self-healable and elastic polyurethane (PU) nanomesh and supramolecular ionic matrix with a high modulus ratio yet matched refractive indices. Self-healable PU is composed of polytetramethylene ether glycol as the soft segment and isophorone diisocyanate/bis(4-hydroxyphenyl)disulfide as the hard segments (Fig. 1c and Supplementary Figs. 1, 2). Aromatic disulfides with highly efficient S-S metathesis were embedded in the hard segment on purpose to provide room temperature self-healability, which is critical for the autonomous healing of PU nanofibers when embedded in the hybrid ionic skin^25^. The cast PU film with a high Young′s modulus of 2.8 MPa slowly self-healed at room temperature for 24 h (Supplementary Fig. 3). PU nanomesh was freshly prepared via electrospinning, which is highly elastic with a structurally deformable fibrous network, yet did not lose its inherent room temperature self-healability as evidenced by the gradual fusion of nanofibers within 36 h (Fig. 2a and Supplementary Figs. 4, 5).

Self-healable supramolecular ionic matrix was prepared by slowly evaporating the viscous aqueous solution of poly(acrylamide-*co*-acrylic acid) (P(AAm-*co*-AA)), hyaluronic acid (HA), and CaCl_2_ in the air to reach final moisture equilibrium (Fig. 1c). Among them, P(AAm-*co*-AA) containing ∼80 wt% AAm acts as the flexible chain for gaining entropic elasticity, and high-concentration CaCl_2_ as the hygroscopic and ionic crosslinking agent. Increasing CaCl_2_ content from 20 wt% to 35 wt% would simultaneously increase the equilibrium water content, ionic conductivity, and stretchability of the resulting ionic matrix, yet elastic recovery and tensile strength were largely reduced (Supplementary Fig. 6). We solved this problem by incorporating a very small amount of semi-rigid HA (1 wt% with respect to P(AAm-*co*-AA)) to further strengthen matrix’s conformational resilience while the conductivity, modulus, and stretchability were almost unaffected (Supplementary Figs. 7, 8). The resulting ionic matrix equilibrated at RH 60% could be reversibly stretched to 1680% strain with a rather low Young′s modulus of 0.3 MPa (1/11 that of PU nanofiber). Notably, ionic matrix contains two main physical crosslinks, i.e., strong hydrogen bonds among the adjacent amide, COOH, and OH groups of PAA and HA, and weak Ca^2+^-mediated ionic complexation with the deprotonated COO^−^^26^. The role of CaCl_2_ in interchain crosslinking was confirmed by rheological tests that the addition of CaCl_2_ transformed P(AAm-*co*-AA)/HA viscous solution to an elastic gel (Supplementary Fig. 9). The strain rate-dependent tensile curves and X-ray diffraction profile of ionic matrix suggest its supramolecular amorphous nature (Supplementary Figs. 10, 11). Moreover, a precut ionic matrix fully healed the scar in 1 h upon incubation at RH 80%, demonstrating its full self-healability owing to the water-plasticized supramolecular network (Supplementary Fig. 12). In addition, cytotoxicity test on HeLa cells proves that the ionic matrix has very good biocompatibility and is thus suitable for on-skin electronic applications (Supplementary Fig. 13).

The slow self-fusion of PU nanomesh allowed easy infiltration of the shear-thinning liquid precursor of the ionic matrix (Supplementary Fig. 14), and subsequent air-drying produced the final hybrid ionic skin with a bicontinuously interlocked structure. The resulting hybrid ionic skin with a thickness of 130 μm appears transparent with ∼80% transmittance in the visible range (Fig. 2b), which is due to the almost equal refractive indices of the ionic matrix (*n* = 1.496) and PU (*n* = 1.503) as well as their uniform hybridization (Supplementary Fig. 15)^27^. As demonstrated by the cross-sectional scanning electron microscope (SEM) image, hybrid ionic skin possesses a sandwich-like structure exposing two thin layers of neat ionic matrix outside; upon stretch, the three-layer structure synergistically deformed with no apparent interlayer delamination or fiber pulling-out (Fig. 2c and Supplementary Fig. 16). This suggests that nanomesh scaffold and ionic matrix are closely interlocked with strong interfacial adhesion (estimated interfacial toughness ∼820 J m^−2^; Supplementary Fig. 17).

### Strain-adaptive stiffening behavior

Strain-stiffening represents one of nature’s key defense mechanisms, which protects skin from accidental damage by malicious stretching while maintaining the initial softness^28^. By mimicking the nanofibrous structure of human skin, our hybrid ionic skin exhibits very similar behavior. As shown in Fig. 2d, the observed J-shaped true stress-strain curves show that hybrid ionic skin rapidly stiffened as compared to PU nanomesh and neat ionic matrix. The calculated differential modulus (∂*σ*_true_/∂*λ*; *σ*_true_ is the true stress, and *λ* the deformation ratio) of hybrid ionic skin first slightly decreased from 1.8 to 1.6 MPa at 75% strain, and then immensely increased to 67.5 MPa at 680% strain in a sigmoid manner, corresponding to ∼37 times stiffness enhancement. As a comparison, the stiffness enhancement factors of PU nanomesh and ionic matrix are much lower (27 and 10 times, respectively). This is mainly because, as stretched, the load-bearing elements of hybrid ionic skin gradually transform from soft ionic matrix to highly stressed and aligned PU nanofibers (Supplementary Fig. 18). As observed in the case of PU nanomesh, the probable strain-induced crystallization of PU soft segments may also contribute to the stiffening response^29^.

The nonlinear strain-stiffening behavior of hybrid ionic skin was highly reproducible in cyclic tensile tests (Supplementary Fig. 19). More importantly, unlike previously reported strain-stiffening supramolecular elastomers^9,26^, the nonlinear stress-strain curves of hybrid ionic skin were almost unaffected by strain rates (Supplementary Fig. 20), which coincides with human skin^30^ more precisely and further verifies the dominating role of PU nanomesh in governing the stiffening response. To demonstrate this strain-stiffening behavior, we applied increasing loads on a strip of hybrid ionic skin (Fig. 2e and Supplementary Movie 1). A small load of 50 g led to immediately a long elongation (159%) of the strip; with further increasing the load to 600 g, the increased elongations became less and less, corresponding to an immensely stiffened sample. Notably, the present strategy is also applicable to other ionic matrices such as PAA-based ionogel and ionoelastomer, which both showed apparent J-shaped strain-stiffening behavior after hybridizing with PU nanomesh (Supplementary Fig. 21).

### Puncture, tear, and fatigue resistances

The nanofibrous structure also imparts very high puncture, tear, and fatigue resistances to the hybrid ionic skin. As shown in Fig. 2f, hybrid ionic skin tolerated much higher puncture forces than the other two control samples (commercial 3 M VHB tape and neat ionic matrix). The toughness of hybrid ionic skin was calculated to be 24.4 MJ m^−3^, superior to the ionic matrix (6.7 MJ m^−3^) and many other reported tough hydrogels/elastomers (Supplementary Table 2 and Supplementary Fig. 22). Moreover, the notched hybrid ionic skin exhibits superb crack tolerance in the tearing process with a maximum strain of 410% (Supplementary Movie 2), and the calculated fracture energy (*Γ*) from the single-edge notch tension (SENT) method is as high as 16.3 kJ m^−2^ (Fig. 2g and Supplementary Fig. 23), 5.3–8.6 times higher than human skin (1.7–2.6 kJ m^−2^) and 6.8 times higher than the ionic matrix (2.1 kJ m^−2^) (Supplementary Fig. 24). The high resistance of hybrid ionic skin to both puncturing and tearing is reasonable since the embedded PU nanofibers create large force transfer lengths through high fiber/matrix modulus ratios and thus blunt cracks from further propagation (Supplementary Fig. 25)^31–33^.

For elastomers, the high resistance to crack growth under monotonic load (characterized by *Γ*) does not always mean the high crack resistance under cyclic loads (characterized by fracture threshold *Γ*_0_). Actually, most load-bearing elastomers are tough but fatigue-prone with a much lower *Γ*_0_ than *Γ*^34^. For example, the well-known tough polyacrylamide-alginate hydrogel has a very high fracture energy of ∼10 kJ m^−2^ yet its fatigue threshold is only 53 J m^−2^ ^35^. However, in hybrid ionic skin, the presence of high-energy PU nanofibers greatly increases the fracture energy per unit area, and thus significantly improves the fatigue resistance. We performed cyclic tensile tests on the pre-notched specimens to evaluate fatigue resistance. The tensile strength of the notched ionic matrix decreased rapidly before fracturing at 200% cyclic strain, and a crack passed through the whole material after 105 cycles (Supplementary Fig. 26). In contrast, hybrid ionic skin could resist 10,000 cycles without obvious crack growth (Fig. 2h). The calculated fatigue threshold of hybrid ionic skin is ∼2950 J m^−2^, about 40 times higher than that of the ionic matrix (∼69 J m^−2^) (Fig. 2i and Supplementary Figs. 27, 28). For comparison, we plotted the fracture energies and fatigue thresholds of a few reported anti-fatigue hydrogels and elastomers in Fig. 2j (data in Supplementary Table 2). Note that, all the samples locate below the *Γ*_0_-*Γ* diagonal line with equal values. Apparently, our hybrid ionic skin with a considerably high fatigue threshold ranks into the top-class fatigue-resistant load-bearing elastomers. The fatigue resistance of hybrid ionic skin was further supported by polarized optical microscope (POM) observations of stress distribution at the notched tip (Fig. 2k). As stretched to 100% strain, very slight stress concentration along the stretching direction could be observed at the notch front with a high-order interference color. Further stretching to larger strains did not extend the crack, and stress concentration had been fully dissipated into bulk material, suggesting efficient crack passivation.

### Self-healability, adhesion, and environmental stability

**Fig. 3:**
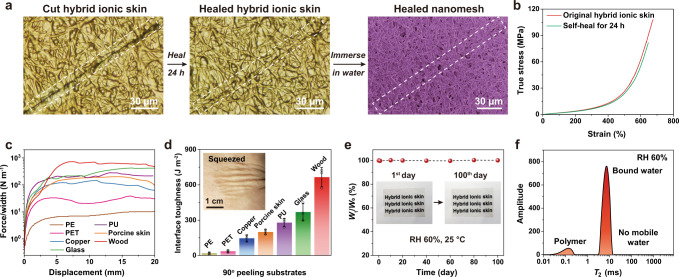
Self-healability, adhesion, and environmental stability of hybrid ionic skin. **a** Optical micrographs of a cut hybrid ionic skin before and after healing at RH 80% for 24 h, and SEM image of healed nanomesh scaffold after washing away ionic matrix. **b** True stress–strain curves of the original and self-healed hybrid ionic skins. **c** Measured peeling forces per width of hybrid ionic skin adhered on various substrates. **d** Corresponding interfacial toughness (inset: a hybrid ionic skin conformally attached to human skin). **e** Water content changes of hybrid ionic skin within 100 days at RH 60%, 25 °C. **f** Low-field ^1^H NMR spectrum of ionic matrix equilibrated at RH 60%. Data in **d** are presented as mean values ± SD, *n* = 3 independent hybrid ionic skins. Source data are provided as a Source Data file.

Benefiting from the self-healability of the two parent components, hybrid ionic skin can autonomously repair fractures on its own. A deep cut that damaged both nanomesh and ionic matrix could be mostly eliminated upon incubation at RH 80% for 24 h (Fig. 3a and Supplementary Movie 3). This event took place through two concurrent healing processes: one is the self-healing of PU nanofibers through dynamic disulfide bond exchange, and the other is the healing of ionic matrix as the mobile water molecules at high humidity would weaken and reconfigure all the physical crosslinks. To prove that PU nanofibers indeed healed, we immersed the healed sample in water to wash away the ionic matrix. As observed by SEM, the cut PU nanomesh rejoined together, suggesting successful self-healing. The healed hybrid ionic skin shows very similar nonlinear stress-strain curve to the original sample, and healing efficiency can reach 85% as evaluated by tensile strength (Fig. 3b). The incomplete self-healing may arise from the poor re-alignment of cut PU nanofibers upon jointing. Nonetheless, the ion-conducting properties could be quickly healed in several cycles of cut and healing processes (Supplementary Fig. 29). As a contrast, although various fiber meshes, 3D printed scaffolds, and fabrics have been previously reported to be hybridized with hydrogels or elastomers, none of these composite materials are both highly stretchable and self-healable due to the inherent rigidity and irreparability of the embedded fiber networks (see the comparison in Supplementary Table 3).

Adhesion is crucial for ionic skins to build intimate human-machine interfaces^36^. The exposed ionic matrix layers of hybrid ionic skin contain abundant functional groups involving polar amides and COOHs as well as ionic COO^-^ and Ca^2+^, which would facilitate strong interfacial interactions with various substrates (Supplementary Fig. 30)^26^. In addition, the dangling chains of the ionic matrix may penetrate the micropores of rough substrate surfaces forming mechanically interlocked structures, also beneficial to improving adhesion performance. With all these benefits, hybrid ionic skin exhibits very strong adhesion to a variety of substrates, including common plastics, metals, elastomers, porcine skin, glass, and wood. The interfacial toughness of hybrid ionic skin on porcine skin and wood can reach as high as 200 and 660 J m^−2^, respectively, as evaluated by 90^o^ peeling tests (Fig. 3c, d). In particular, the adhesive hybrid ionic skin is very complaint to conformally deform with human skin, demonstrating its great potential to be applied in epidermal electronics.

For water-containing ionic skins, environmental stability is a major challenge as water may either evaporate at low humidities or freeze at subzero temperatures^37^. Here we show that the hygroscopic ion-rich nature of hybrid ionic skin imparts very high environmental stability. The introduced CaCl_2_ can not only coordinate with trapped water to improve water retention ability, but also depress the freezing point by improving the colligative property^38^. Therefore, hybrid ionic skin could keep the initial quality and shape almost unchanged after storage in an open environment (RH 60%, 25 °C) for 100 days (Fig. 3e). Even if completely dried, its mechanical property and ionic conductivity were fully restored after incubating at RH 60% for 1 h (Supplementary Figs. 31a, b). Stored at fixed humidities, the ionic conductivity of hybrid ionic skin remained stable for a long time (Supplementary Fig. 31c). Differential scanning calorimetry (DSC) did not detect any water freezing event of hybrid ionic skin down to −90 °C, indicating its excellent anti-freezing property (Supplementary Fig. 32). We evaluated the mobility of water molecules in the ionic matrix by low-field ^1^H NMR. As shown in Fig. 3f, there is only bound water in the ionic matrix, which accounts for its good anti-freezing performance since freezing mainly relies on mobile water.

### Enhanced strain sensation and applications

**Fig. 4:**
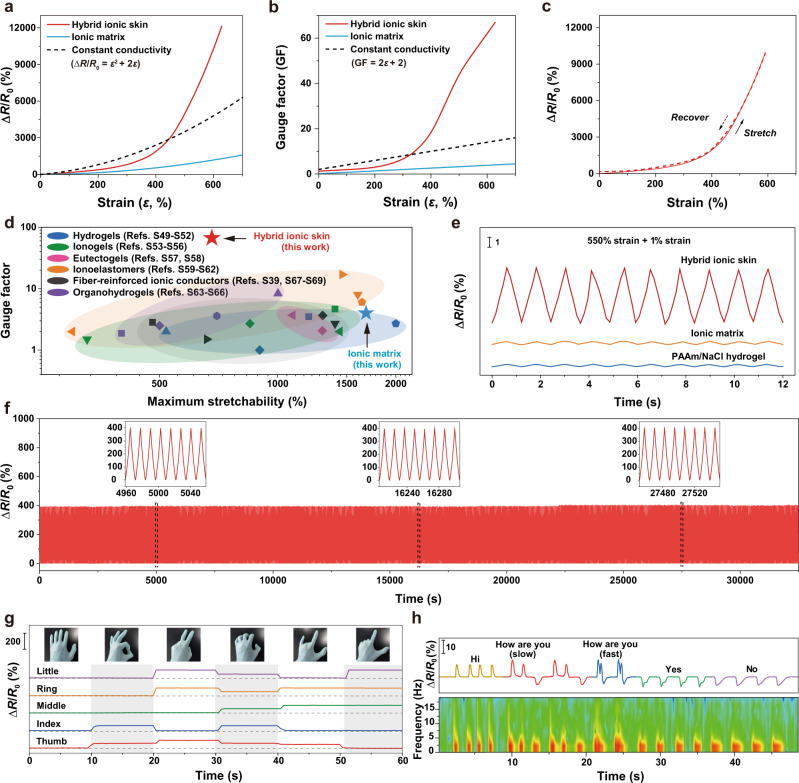
Enhanced strain sensation of hybrid ionic skin. **a** Strain-dependent resistance changes of hybrid ionic skin, ionic matrix, and conductivity-constant conductors. **b** Corresponding gauge factor (GF) changes. **c** Reversible resistance changes of hybrid ionic skin as stretched and recovered. **d** Comparison of GF values and maximum stretchability among hybrid ionic skin and other intrinsically stretchable ionic conductors. **e** Comparison of resistance changes among hybrid ionic skin, ionic matrix, and PAAm/NaCl hydrogel by superimposing 1% strain on fixed 550% strain. **f** Real-time response by stretching to 200% strain for 2500 cycles. **g** Multichannel monitoring of gestures with five adhered hybrid ionic skin sensors. **h** Voice recognition with an attached hybrid ionic skin sensor on a volunteer’s throat. Source data are provided as a Source Data file.

The movable ions and excellent elasticity provide hybrid ionic skin with strain-sensing properties. Figure 4a shows that the electrical resistances of ionic matrix and hybrid ionic skin both increased with strain changes, typical for stretchable ionic conductors. However, hybrid ionic skin exhibits a surprisingly higher mechanoelectrical response than the ionic matrix and conductivity-constant conductors (e.g., ionic liquids, liquid metals, etc.; Δ*R*/*R*_0_ = *ε*^2^ + 2*ε*; Δ*R* is the resistance change (= *R*−*R*_0_), *R* and *R*_0_ the strain-dependent and initial resistances) at large strains. This is quite unusual, as most reported ionic conductors show only a moderate mechanoelectrical response (i.e., always lower than the conductivity-constant curve)^39^. The calculated gauge factor (GF = ∂(Δ*R*/*R*_0_)/∂*ε*) of hybrid ionic skin can reach as high as 66.8 at 630% strain, significantly higher than the ionic matrix (GF = 4.0) and conductivity-constant conductors (GF = 14.6) at the same strain (Fig. 4b). In addition, the high mechanoelectrical response of hybrid ionic skin was totally reversible (Fig. 4c). We compared the maximum GF and stretchability of hybrid ionic skin with a few previous intrinsically stretchable ionic conductors and fiber-reinforced ionic conductors (Fig. 4d and Supplementary Table 4). Apparently, the strain sensitivity of hybrid ionic skin with a record-high GF of 66.8 surpasses all the reported stretchable ionic conductors. In contrast, the conventional strategies to improve the GFs of ionic conductors mainly rely on introducing electronically conductive fillers, which often sacrifice the material’s transparency, softness, and stretchability^40^.

To demonstrate the enhanced strain sensation, we reversibly stretched hybrid ionic skin fixed at 550% strain in a very small strain amplitude of 1%. Neat ionic matrix and polyacrylamide/NaCl hydrogel were chosen as the representatives of common ion-conducting strain sensors. The sensing performance of hybrid ionic skin is obviously better than the other two control samples (Fig. 4e). Besides, superimposing small strains (1%) on increasing fixed strains (100%, 300%, 500%) produced also reliable electrical response (Supplementary Fig. 33). Furthermore, hybrid ionic skin-based sensor is highly durable, as supported by the stable response over 2500 stretching cycles to a fixed strain of 200% (Fig. 4f). The electrical response is timely with a typical response time of 165–190 ms (Supplementary Fig. 34). Even after stretching for 10,000 cycles, the strain-dependent resistance response was still reproducible, highlighting the fatigue resistance of hybrid ionic skin (Supplementary Fig. 35).

As wearable sensors, five hybrid ionic skins attached to five fingers could simultaneously monitor and distinguish different gestures according to distinct signals at different channels (Fig. 4g). The attached sensor could also monitor real-time human motions such as knee bending, tiny muscle vibrations during the speech, and blood pressure pulse on the wrist (Fig. 4h and Supplementary Figs. 36, 37). In addition to strain, the hybrid ionic skin could also act as a multifunctional sensor for detecting temperature and moisture variations as well as extracting electrophysiological signals. For example, contact-free temperature variations and moisture-related human respiration were easily detected by the hybrid ionic skin (Supplementary Figs. 38–41). To decouple strain and temperature sensing, a hybrid ionic skin-based capacitive sensor was further configured, as the capacitance is only sensitive to strain changes (Supplementary Fig. 42). Besides, the hybrid ionic skin as an on-skin conformal electrode was able to extract high-resolution electrocardiogram (ECG) and electromyogram (EMG) information (Supplementary Figs. 43, 44).

### Mechanism discussion for enhanced strain sensation

**Fig. 5:**
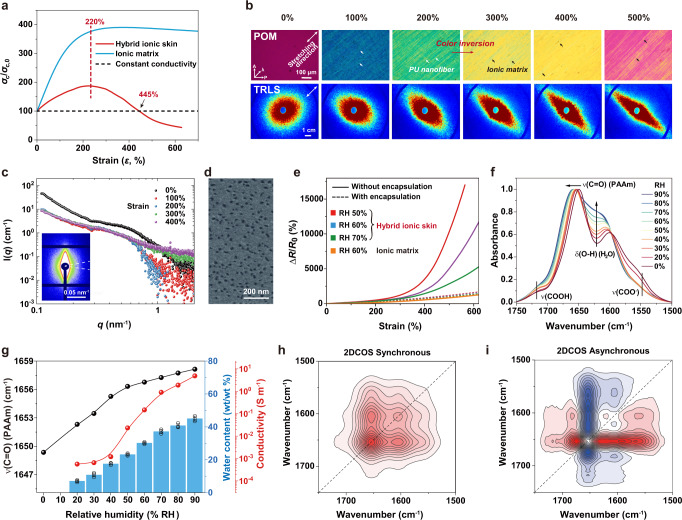
Mechanism analysis for the enhanced strain sensation of hybrid ionic skin. **a** Strain-dependent ionic conductivity changes (*σ*_c_/*σ*_c,0_) of hybrid ionic skin, ionic matrix, and conductivity-constant conductor. **b** POM images (top) and 2D TRLS patterns (bottom) of hybrid ionic skin with increasing strains. **c** SAXS scattering intensity plots of hybrid ionic skin at increasing strains (inset: 2D pattern at 200% strain showing the horizontal integral direction). **d** SEM image of vacuum-dried ionic matrix. **e** Strain-dependent resistance changes of hybrid ionic skin and ionic matrix equilibrated at different humidities and with/without VHB encapsulation. **f** IR spectra of ionic matrix equilibrated at different humidities. **g** Humidity-dependent *v*(C = O) (PAAm), water contents, and ionic conductivities. **h**, **i** 2DCOS synchronous (**h**) and asynchronous spectra (**i**) generated from time-resolved moisture-absorbing IR spectra (Supplementary Fig. 47). In 2DCOS maps, red colors represent positive intensities, while blue colors indicate negative ones. Data in **g** are presented as mean values ± SD, *n* = 3 independent ionic matrices. Source data are provided as a Source Data file.

To clarify the mechanism of the substantially enhanced strain sensation of hybrid ionic skin, we first transformed the resistance changing curves in Fig. 4a to conductivity changes via the equation (*σ*_c_/*σ*_c,0_ = *λ*^2^/(*R*/*R*_0_); *σ*_c_ and *σ*_c,0_ are the strain-dependent and initial conductivities, respectively)^39^, which reflect intrinsic structural changes without considering shape parameters. As shown in Fig. 5a, the neat ionic matrix exhibited a gradual conductivity increase as stretched and reached a plateau at ∼220% strain, typical for ionic conductors with chain alignment-promoted ion transportation behavior. Interestingly, hybrid ionic skin demonstrated a maximum conductivity also at 220% strain, which then drastically decreased till break. At 445% strain, the ionic conductivity even equals that at the undeformed state. These observations reveal that the ion-conducting behavior of hybrid ionic skin is dominated by the intrinsic deformation of the ionic matrix before 220% strain, and afterward, by the synergistic deformations of nanomesh scaffold and matrix resulting in sharply reduced conductivities.

The proper understanding of the mutual interplay between PU nanomesh and the ionic matrix is the key to uncovering the unusual conductivity reduction at large strains. We observed the strain-dependent orientation changes of hybrid ionic skin with POM, time-resolved light scattering (TRLS), and small-angle X-ray scattering (SAXS) (Fig. 5b, c). 2D TRLS pattern gradually transformed from roughly round to date palm-like shapes with increasing strains, corresponding to the anisotropic stretching of the original randomly aligned nanofibers at the micrometer scales. Such a stretch-induced fiber orientation was evidenced from POM observations with interference colors gradually turning to higher retardations. Noteworthily, a unique color inversion was observed between 200 and 300% strains, which is ascribed to the significant increase in the area occupied by fibers due to fiber bundling^41^. 2D SAXS pattern at 200% strain appeared anisotropic meridional scattering, indicating stretch-induced high orientation also at the nanometer scales. The SAXS curves (obtained by integrating along the horizontal direction) at 0–200% strains showed a plateau arising from the ionic matrix (Supplementary Fig. 45), corresponding to a nanoporous structure with a radius of gyration (*R*_g_) of about 3.1 nm (pore diameter >8 nm in the wet state). When strain increased to 300% and above, the plateau disappeared, suggesting that the nanoporous structure became denser. Such a nanoporous structure was also evidenced from the SEM observation of vacuum-dried ionic matrix which showed numerous nanopores with a mean size of 21 nm (Fig. 5d and Supplementary Fig. 46). The nanopores may be caused by the crosslinking inhomogeneities arising from two distinct physical crosslinks. This also indicates that, although water is confined in the ionic matrix, salt-promoted moisture breathing may readily occur across nanopores through concurrent liquid diffusion, interfacial liquid-gas sorption, and vapor transport^42^. All the above results direct to a process that after 200% strain, highly aligned PU nanofibers start to squeeze soft ionic matrix resulting in denser nanopores.

To clarify what occurred to the ionic matrix, we measured the strain-dependent resistance changes of hybrid ionic skin and ionic matrix equilibrated at different humidities with and without encapsulation (Fig. 5e). The resistance changing curves of all the encapsulated samples coincided with each other, demonstrating a typical moderate mechanoelectrical behavior. In contrast, the unencapsulated hybrid ionic skin exhibited enhanced strain sensation, and the lower environmental humidity was, the higher resistance increased. Neat ionic matrix did not show such a difference with and without encapsulation. It is suggested that moisture exchange may exist between hybrid ionic skin and atmospheric environment in the stretching process. In consideration of its nanoporous structure, the ionic matrix may breathe out moisture forced by the stretched and bundled PU nanomesh, and vice versa.

To support the hypothesized breathing effect, we monitored the infrared (IR) spectra of the ionic matrix equilibrated at different humidities. The internal interactions reflected by IR peak positions exhibited very high sensitivity to humidity changes (Fig. 5f). As humidity increased, the O-H bending vibration of water was gradually overlapped into the whole spectra, and the amide C=O stretching of PAAm shifted to higher wavenumbers, suggesting weakened self-associations. Such a humidity-dependent peak shift can be directly linked to water content and ionic conductivity changes (RH 20%: 1652.3 cm^−1^, 7 wt%, 5.5 × 10^−4^ S m^−1^; RH 90%: 1658.1 cm^−1^, 45 wt%, 4.8 S m^−1^) (Fig. 5g). We further performed two-dimensional correlation spectroscopy (2DCOS) analysis based on the time-resolved moisture-absorbing IR spectra (Supplementary Fig. 47) to extract more subtle information about interaction changes. 2DCOS contains a pair of synchronous and asynchronous spectra (Fig. 5h, i), and from their cross-peak signs, event sequence can be deduced to help infer the sensitivities of different groups to an external stimulus. According to Noda’s rule and order determination table (Supplementary Table 5), the obtained sequence is (→ means prior to or earlier than), 1547 → 1687 → 1714 → 1574 → 1674 → 1622 → 1603 → 1655 cm^−1^; that is, *v*(COO^−^) (Ca^2+^ complexed) → *v*(C=O) (free PAAm) → *v*(COOH) (PAA) → *v*(COO^−^) (free) → *v*(C=O) (weakly H-bonded PAAm) → *δ*(O-H) (H_2_O) → Amide II → *v*(C=O) (strongly H-bonded PAAm). Such order indicates that the moisture-breathing process of the ionic matrix is driven by Ca^2+^:COO^−^ complexation changes, which are most prone to be attacked by environmental water molecules.

Altogether, based on the above analyses, the enhanced strain sensation of hybrid ionic skin should be attributed to the nanofiber tension-induced reversible moisture breathing of the ionic matrix. The internal compressive pressure generated from bundled nanofibers forces hygroscopic ionic matrix to desorb a certain amount of water molecules into the atmospheric environment, which lowers the whole ionic conductivity. Such a fiber tension-induced squeezing effect is reminiscent of the compressive force exerted by stretched tissue fibrils leading to the volume shrinkage and fluid exudation of the matrix in clots and tendons^41,43^. At the molecular level, the breathing of ionic matrix is found to be mainly driven by the interaction changes of highly water-sensitive Ca^2+^:COO^−^ complexation, which meanwhile influences charge diffusion through Cl^−^. The reduced mobility of the negative charge carrier (Cl^−^) upon a conformational pressure was well supported by molecular dynamics simulation and thermoelectric results (Supplementary Figs. 48–51).

## Discussion

In this work, by mimicking the healable nanofibrous composite structure of human skin, we demonstrate an artificial hybrid ionic skin by embedding a randomly aligned, high-energy, and self-healable PU nanomesh into the soft supramolecular ionic matrix. Such a nanofibrous structure endows hybrid ionic skin with the unrivaled combination of a few intriguing properties that are unique to human skin yet difficult to realize for its artificial analogs, including self-healing (efficiency ∼85%), softness (modulus ∼1.8 MPa), strain-adaptive stiffening (37 times stiffness enhancement), stretchability (680%), fatigue resistance (*Γ*_0_ ∼ 2950 J m^−2^), ionic conductivity (∼0.11 S m^−1^ at RH 60%), and superior strain sensation. Intriguingly, a record-high strain-sensing gauge factor of 66.8 is realized, which is ascribed to the nanofiber tension-induced moisture breathing effect of hygroscopic ionic matrix driven by water-sensitive ionic complexations. In combination with its transparency, adhesiveness, and ambient stability, the hybrid ionic skin demonstrates great potential as highly sensitive and durable sensors to be applied in wearable electronics and human-machine interfacing, etc. We anticipate that the proposed concept will be easily extended to fabricate other robust ion-conducting materials with a variety of material combinations.

## Methods

### Materials

Acrylamide (AAm), acrylic acid (AA), *N*,*N*′-methylenebisacrylamide (MBAA), 1-ethyl-3-methylimidazolium ethyl sulfate (EMI ES), and isophorone diisocyanate (IPDI) were purchased from TCI Chemical. Calcium dichloride (CaCl_2_) was obtained from Rhawn Reagent. Hyaluronic acid (HA) was purchased from Ourchem Biotechnology (China). Polytetramethylene ether glycol (PTMEG, *M*_n_ = 1000 g mol^−1^), dibutyltin dilaurate (DBTDL), polyacrylic acid (PAA, *M*_w_ ≈ 250,000 g mol^−1^, 35 wt% in H_2_O), and [2-(acryloyloxy)ethyl]trimethylammonium chloride (DMAEA-Q) were purchased from Sigma-Aldrich. Bis(4-hydroxyphenyl) disulfide and *N*,*N*-dimethylacetamide (DMAc) were purchased from Energy Chemical. Tetrahydrofuran (THF) and potassium persulfate (KPS) were purchased from Adamas Reagent. The photoinitiator, 2-oxoglutaric acid, was obtained from Fluka Chemical. All chemicals were used as received without further purification. Ultrapure water (Milli-Q, 18 MΩ cm) was used for all the related experiments.

### Synthesis of self-healing P(AAm-*co*-AA)/HA/CaCl_2_ ionic matrix

The copolymer, poly(acrylamide-*co*-acrylic acid) (P(AAm-*co*-AA), 80 wt% acrylamide), was synthesized by the free radical polymerization of the precursor solution containing AA, AAm, and initiator KPS (0.2 wt % with respect to the total mass of monomer) with a total monomer concentration of 5 wt% at 70 °C for 6 h. The resulting highly viscous liquid was further dialyzed by ultrapure water to remove unreacted impurities. After that, the solvent was removed by freeze-drying to obtain a white powder. The weight-average molecular weight (*M*_w_) of the obtained copolymer is 4.6 × 10^4^ g mol^−1^ with the polydispersity of 2.39, as measured by aqueous GPC (Waters 1525; eluent: 0.2 M NaNO_3_, 0.01 M NaH_2_PO_4_, pH 7).

Then different mass fractions of CaCl_2_ and HA were added to 15 wt% P(AAm-*co*-AA) solution to adjust the ionic matrix’s composition. The resulting viscous gel precursor was then poured into a PTFE mold and evaporated in a constant temperature & humidity chamber (RH 60%, 25 °C) to form the final self-healing ionic matrix. For infiltrating nanomesh to prepare hybrid ionic skin, the precursor was adjusted to a total polymer concentration of 10.8 wt% (containing 1 wt% HA) and CaCl_2_ concentration of 5.7 wt%.

### Synthesis of self-healing PU

Self-healing PU containing PTMEG as the soft segment, and IPDI/bis(4-hydroxyphenyl) disulfide as hard segments were prepared according to the literature^25,44^. In brief, PTMEG (14.5 g) was added into a dried three-neck reactor fitted with a mechanical stirrer and then heated in an oil bath at 100 °C under vacuum for 1 h to remove residual moisture, followed by cooling to 70 °C. IPDI (6.77 g) and DBTDL (0.05 g) completely dissolved in DMAc (5 mL) were slowly added into the flask and stirred for 2 h under argon atmosphere. After that, bis(4-hydroxyphenyl) disulfide (3.63 g) as the chain extender dissolved in DMAc (10 mL) was directly added to the reactor (25 °C), which was then heated to 40 °C for 18 h. The weight-average molecular weight (*M*_w_) of the obtained self-healing PU is 1.07 × 10^5^ g mol^−1^ with the polydispersity of 2.23, as evaluated by GPC (Agilent PL-GPC50; eluent: THF).

### Preparation of PU nanomesh

PU nanomesh was prepared with a modified electrospinning method according to the literature^44^. Typically, the obtained self-healing PU was first dissolved in the mixture of THF and DMAc (30/70, wt/wt) under mild stirring for 24 h to obtain a 20 wt% transparent solution. Then, the PU solution was electrospun at an applied voltage of 10.3 kV, flow rate of 0.7 mL h^−1^, the working distance of 11 cm, and drum collector (covered with aluminum foil) speed of 300 rpm. The fiber collector was swung back and forth to make the thickness of PU nanomesh uniform. Environmental temperature and relative humidity were controlled at 18 ± 2 °C and 40 ± 5%, respectively.

### Preparation of PU nanomesh-coupled hybrid ionic skin

The freshly prepared PU nanomesh with a thickness of ∼110 μm was treated by O_2_ plasma for 30 s to make its surface hydrophilic. Afterwards, the nanomesh was cut and sandwiched by two rectangular hollow silicone spacers (100 μm thick, one side adhesive), which were then immersed in the pre-gel solution of ionic matrix and degassed in a vacuum desiccator. Subsequently, the whole device was brought back to the atmosphere, along with the pre-gel solution penetrating into the porous network of electrospun nanomesh assisted by atmospheric pressure. The vacuum-assisted penetration process was repeated three times. Finally, the resulting wet composite membrane was placed in a constant temperature & humidity chamber (RH 60%, 25 °C) for evaporation to form the final hybrid ionic skin. The sample was repeatedly stretched to 500% strain 10 times to reach a stable interlocked state before use.

### Preparation of PAAm/NaCl hydrogel

Chemically crosslinked PAAm/NaCl hydrogel was synthesized via radical polymerization at room temperature^45^. A precursor solution was prepared by adding chemical cross-linker MBAA (0.03 mol % with respect to AAm) and photoinitiator 2-oxoglutaric acid (0.1 mol % with respect to AAm) to a solution containing 4 M AAm and 0.01 M NaCl. The precursor solution was then polymerized under 365 nm UV irradiation for 30 min to form the hydrogel.

### Characterizations

Rheological properties were measured at different frequencies using a Thermo Scientific HAAKE MARS 60 modular advanced rheometer. X-ray diffraction (XRD) was measured on Bruker D8 diffractometer using Ni-filtered Cu Kα radiation (40 kV, 40 mA). The microscopic structures were observed by a field-emission scanning electron microscope (FE-SEM, Regulus 8230) and a POM (Olympus BX53-P). The optical transmittance ranging from 400 to 800 nm was measured on UV-vis spectrometer (Lambda 950, PerkinElmer). DSC was conducted on TA Q250 at a heating/cooling rate of 5 °C min^−1^ under N_2_ atmosphere. ^1^H NMR spectrum of self-healing PU was recorded on Bruker AV-400 using CDCl_3_ as the solvent. The refractive indices of various materials were measured by a hand-held digital refractometer (PAL-RI; ATAGO) at room temperature. The stretch-induced macroscopic orientation changes of hybrid ionic skin at the micrometer scale were observed in situ on a self-made time-resolved light scattering (TRLS) instrument. Low-field ^1^H NMR spectrum of an ionic matrix was measured on a VTMR20-010V-I NMR analyzer (Suzhou Niumag Analytical Instrument Corporation, China). Unless otherwise specified, material properties were all measured at ambient conditions (RH 60 ± 1%, 25 °C).

### Mechanical characterizations

All the tensile experiments were carried out using a UTM 2103 universal testing system with a 100 N load cell. Typically, a strip sample (3 mm wide) was clamped with an initial gauge length of 9 mm. Since sample dimensions constantly change during deformation, true stress was also used for strength evaluation, calculated by multiplying nominal stress by the deformation ratio based on the incompressible assumption. Tensile toughness was calculated from the integrated area below the nominal stress-strain curve up to the point of fracture.

### Peeling adhesion tests

90^o^ peeling adhesion tests were performed on a vertical dynamometer (ESM303, MARK-10) at a constant peeling speed of 50 mm min^−1^. The interfacial toughness was determined by dividing the plateau force by sample width.

### Single-edge notch tension (SENT) tests

For SENT tests, two different samples, notched and unnotched, were used to measure the fracture energy *Γ*^46^. The samples were cut into a rectangular shape. For notched samples, a notch was made in the middle of one side by using a sharp razor. The distance between two clamps was fixed to 9 mm, and tensile speed was kept at 18 mm min^−1^. The fracture energy (*Γ*) was calculated by the following equation:

1 $$\varGamma {{\mbox{=}}}\frac{6{Wc}}{\surd {\lambda }_{c}}$$

where *λ*_*c*_ is the fracture deformation ratio of notched sample (*λ*_*c*_ = *ε*_*c*_ + 1), *c* represents the length of the notch, and *W* is the strain energy calculated by integration of the stress versus strain of unnotched specimens with the same dimensions stretched to the *ε*_c_ strain.

### Fatigue tests

Fatigue threshold was measured by the similar single-edge notch method at ambient conditions (RH 60 ± 1%, 25 °C)^12,15^. The used rectangular sample had a width of 3 mm, thickness of 0.13 mm, and gauge length of 8 mm. Notched specimens with precut cracks (∼1/5 of the width) were cyclically stretched at a strain rate of 3.3% s^−1^ under different strains with no pause. The energy release rate (*G*) of notched sample under the *N*th cycle was calculated as follows:

2 $$G(N)=2k(N){{\cdot }}c(N){{\cdot }}W(N)$$

Where *k*(*N*) is a function of strain variation determined by $k(N)=3/\sqrt{{{\varepsilon }}+1}$, *c*(*N*) is the crack propagation length, and *W*(*N*) is the strain energy density of unnotched specimens of the same dimensions stretched to the same strain *ε*. The strain energy density *W*(*N*) of unnotched sample under the *N*th cycle can be obtained using $W{{\mbox{(}}}N{{\mbox{)=}}}{\int }_{0}^{\varepsilon }\sigma {{\mbox{d}}}\varepsilon$.

### Electrical characterizations

The real-time resistance signals of the samples under different strains, temperatures, or humidities were recorded by Tektronix multimeter (DMM 4050) and Keithley multichannel source meter (DAQ6510). During test, electrical connections were made by conductive copper tapes. The heating and cooling cycles were carried out on a temperature-controlled platform (LTS420, Linkam). Prior to test, the sample was sealed on a glass plate with 3 M VHB tape to reduce the influence of humidity on its conductivity. ECG signals were measured using a Heal Force ECG recorder (PC-80B). EMG signals were recorded using the two-channel EMG sensor (ED0136/EDK0056, SICHIRAY). For adhesion and sensing applications, the hybrid ionic skins were attached to several body parts of a volunteer who participated in the experiment approved by the Institutional Biomedical Research Ethics Committee of Shanghai Institutes for Biological Sciences (No. 3011-19-03). All human participants gave written and informed consent before related experiments.

### Calculation of ionic conductivity change

According to Pouillet’s Law, the resistance (*R*) of the ionic matrix can be expressed as *R* = *l*/(*σ*_c_·*A*), where *σ*_c_ is the ionic conductivity, *l* the length, and *A* the cross-sectional area. In the case of a constant volume of ideal elastomer during deformation (*V* = *l*·*A* = *l*_0_·*A*_0_), the ionic conductivity change was calculated as follows:

3 $${\sigma }_{{{{{{\rm{c}}}}}}}/{\sigma }_{{{{{{\rm{c}}}}}},0}=	 [l/(R{{\cdot }}A)]/[{l}_{0}/({R}_{0}{{\cdot }}{A}_{0})]=(l/{l}_{0})/[(A/{A}_{0}){{\cdot }}(R/{R}_{0})] \\=	 {(l/{l}_{0})}^{2}/(R/{R}_{0})={\lambda }^{2}/(R/{R}_{0})$$

where *σ*_c,0_, *R*_0_, *l*_0_, *A*_0_ are the initial conductivity, resistance, length, and cross-sectional area, respectively. The deformation ratio (*λ*) is defined as *λ* = *l*/*l*_0_. Note that, *λ* = *ε* + 1 (*ε*, strain).

### Small-angle X-ray scattering

SAXS measurements were carried out on the SSRF beamline BL16B (Shanghai, China), where the X-ray energy was 10.0 keV and the wavelength was 1.24 Å. During measurement, the samples were placed perpendicular to the beam with the distance from the sample to the detector of 1.87 m to cover the scattering vector *q* ranging from 0.1 to 6 nm^−1^ (*q* is the scattering vector, *q* = (4π/*λ*)sin(*θ*); 2*θ*, the scattering angle). The acquisition time of light scattering patterns was 180 s, and the intensity profiles were obtained by radially averaging SAXS patterns in the horizontal direction.

### Time-resolved moisture-absorbing ATR-FTIR spectra

All the attenuated total reflection Fourier transform infrared (ATR-FTIR) spectra were collected on a Thermo Scientific Nicolet iS50 spectrometer with a diamond ATR crystal as the window material at room temperature. For the time-resolved moisture-absorbing experiment, a fully dry ionic matrix with a thickness of 1 mm was placed on the diamond crystal. Time-resolved ATR-FTIR spectra were recorded with an interval of 1 min (software: Omnic, ver. 7.4) at the ambient condition of RH 60%, 25 °C when the dry ionic matrix gradually absorbed moisture from the air.

### Two-dimensional correlation spectroscopy

The time-resolved moisture-absorbing ATR-FTIR spectra of the ionic matrix from 0 to 50 min were used for performing 2D correlation analysis. The software 2D Shige ver. 1.3 (©Shigeaki Morita, Kwansei-Gakuin University, Japan, 2004–2005) was used for 2D correlation analysis, and OriginPro program, ver. 9.9 was further used to draw contour maps.

### Molecular dynamics simulation

A periodic model of ionic matrix containing one P(AAm-*co*-AA) chain (40 repeating units containing 32 AAm and 8 AA), 20 CaCl_2_, and 80 H_2_O molecules was constructed in the Amorphous Cell module of Materials Studio, ver. 2019. The dynamic structural optimization was performed in the Forcite module (NPT @ 298 K, 0 GPa) for 1000 ps. Increasing conformational pressure to 0.5 GPa was applied to simulate the compressed state of the ionic matrix. Based on the dynamics simulation results, mean square displacement (MSD) was employed to calculate the self-diffusion coefficients of ions under different pressures (*D* = 1/6 slope of MSD curve), and radial distribution functions between O and H atoms of water were used to evaluate interactions among water molecules in the whole system.

### Cytotoxicity tests of ionic matrix

The cytotoxicity of the ionic matrix was assessed with HeLa cells (cervical cancer, purchased from Chinese Academy of Sciences Cell Bank, catalog No. TCHu187) by Cell-Counting-Kit-8 (CCK-8) assays. HeLa cells were seeded in 96-well plates at a density of 1 × 10^4^ cells per well, and cultured in a 5% CO_2_ environment at 37 °C for 24 h. Subsequently, the original medium was replaced with a fresh DMEM containing ionic matrix (final concentration 0–2 mg ml^−1^) for incubation. 100 μL of CCK-8 solution (diluted with DMEM at the ratio of 1:9) was added to each well for 1 h at 37 °C. The absorbance was measured at 450 nm by a microplate reader.

### Statistics and reproducibility

All experiments were repeated independently with similar results at least three times.

### Reporting summary

Further information on research design is available in the Nature Research Reporting Summary linked to this article.

## Supplementary information

Supplementary Information

Description of Additional Supplementary Information

Supplementary Movie 1

Supplementary Movie 2

Supplementary Movie 3

Reporting Summary

## Data Availability

All data supporting the findings of this study are available within this article and Supplementary Information or from the corresponding author upon reasonable request. Source data are provided with this paper.

## Source data

Source Data
