# Supplementary material for: Fatigue-free artificial ionic skin toughened by self-healable elastic nanomesh
Source: Nat Commun. 2022 Jul 29;13:4411. doi: 10.1038/s41467-022-32140-3 (PMC9338060; doi:10.1038/s41467-022-32140-3)
Supplement: Supplementary file 1 — Supplementary Information [file 41467_2022_32140_MOESM1_ESM.pdf]

*Supplementary Information for*

# Fatigue-free artificial ionic skin toughened by self-healable elastic nanomesh

Jiqiang Wang, Baohu Wu, Peng Wei, Shengtong Sun*, and Peiyi Wu*

*Correspondence to: shengtongsun@dhu.edu.cn; wupeiyi@dhu.edu.cn

**This material includes**

**Extended Methods**

**Suppl. Fig. 1.** Synthesis of self-healing PU
**Suppl. Fig. 2.** $^1$H NMR spectrum of self-healing PU
**Suppl. Fig. 3.** Optical micrographs and tensile curves of PU film before and after self-healing
**Suppl. Fig. 4.** SEM images of self-healing PU nanomesh under different strains
**Suppl. Fig. 5.** SEM images of self-healing PU nanomesh at different time
**Suppl. Fig. 6.** Effects of $CaCl_2$ content on the P(AAm-*co*-AA)/$CaCl_2$ ionic conductor
**Suppl. Fig. 7.** Stress-strain curves and ionic conductivities of ionic conductors with/without HA
**Suppl. Fig. 8.** Cyclic stress-strain curves of ionic conductor at increasing strains
**Suppl. Fig. 9.** Rheology of P(AAm-*co*-AA)/HA aqueous solutions with/without $CaCl_2$
**Suppl. Fig. 10.** Strain rate-dependent tensile curves of ionic conductor
**Suppl. Fig. 11.** XRD profile of ionic conductor
**Suppl. Fig. 12.** Optical microscopic images of ionic conductor in the self-healing process
**Suppl. Fig. 13.** Cytotoxicity test of ionic matrix
**Suppl. Fig. 14.** Shear-thinning behavior of ionic conductor precursor
**Suppl. Fig. 15.** Optical microscopic image of hybrid ionic skin
**Suppl. Fig. 16.** Cross-sectional SEM images of hybrid ionic skin under different strains
**Suppl. Fig. 17.** 90$^o$ peeling curve between ionic conductor and PU nanomesh
**Suppl. Fig. 18.** Schematic nonlinear elasticity of hybrid ionic skin
**Suppl. Fig. 19.** Cyclic tensile curves of hybrid ionic skin at a fixed strain of 500%
**Suppl. Fig. 20.** Tensile curves of hybrid ionic skin at different strain rates
**Suppl. Fig. 21.** Tensile curves of PU nanomesh-hybridized ionogel and ionoelastomer
**Suppl. Fig. 22.** Comparison of toughness and Young′s modulus of hybrid ionic skin with reported toughened hydrogels and elastomers
**Suppl. Fig. 23.** SENT-calculated fracture energies with different notch sizes and sample widths
**Suppl. Fig. 24.** Nominal stress-strain curves of unnotched and notched ionic conductor
**Suppl. Fig. 25.** SEM images of the fractured surfaces of notched hybrid ionic skin and ionic matrix
**Suppl. Fig. 26.** Cyclic tensile curves of notched ionic conductor at 200% strain
**Suppl. Fig. 27.** Stress decays of hybrid ionic skin at different energy release rates
**Suppl. Fig. 28.** Crack extension per loading cycle versus energy release rate for ionic conductor
**Suppl. Fig. 29.** Healed ionic conductivity of hybrid ionic skin
**Suppl. Fig. 30.** Schematic illustration of 90° peeling test for hybrid ionic skin

**Suppl. Fig. 31.** Regeneration of hybrid ionic skin and its conductivity stability
**Suppl. Fig. 32.** DSC curve of PU nanomesh and hybrid ionic skin equilibrated at RH 60%
**Suppl. Fig. 33.** Resistance changes of hybrid ionic skin by superimposing 1% strains on fixed strains
**Suppl. Fig. 34.** Response time of hybrid ionic skin sensor
**Suppl. Fig. 35.** Strain-dependent resistance changes before and after stretching for 10,000 cycles
**Suppl. Fig. 36.** Monitoring knee bending with hybrid ionic skin sensor
**Suppl. Fig. 37.** Monitoring human radial pulse by hybrid ionic skin attached on the wrist
**Suppl. Fig. 38.** Temperature-dependent resistance changes of hybrid ionic skin/ionic conductor
**Suppl. Fig. 39.** Temperature-dependent reversible resistance changes of hybrid ionic skin
**Suppl. Fig. 40.** Electrical response of hybrid ionic skin when hot water repeatedly approached
**Suppl. Fig. 41.** Monitoring human respiration by hybrid ionic skin
**Suppl. Fig. 42.** Decoupling strain and temperature sensing by the capacitive sensor
**Suppl. Fig. 43.** ECG signals of a volunteer recorded by hybrid ionic skin electrode
**Suppl. Fig. 44.** EMG signals of a volunteer recorded by hybrid ionic skin electrode
**Suppl. Fig. 45.** SAXS profiles of hybrid ionic skin and PU nanomesh
**Suppl. Fig. 46.** SEM images of vacuum-dried ionic conductor and size distribution of nanopores
**Suppl. Fig. 47.** Time-resolved ATR-FTIR spectra of moisture-absorbing ionic conductor
**Suppl. Fig. 48.** Amorphous cell of ionic matrix for molecular dynamics simulation
**Suppl. Fig. 49.** Computed mean square displacement curves of $Ca^{2+}$ and $Cl^{-}$
**Suppl. Fig. 50.** Radial distribution functions of O next to H atoms of water molecules
**Suppl. Fig. 51.** Open-circuit voltage variation of ionic conductor with temperature gradient

**Suppl. Table 1.** Comparison of combinatory properties of human skin and hybrid ionic skin
**Suppl. Table 2.** Comparison of mechanical performance among hybrid ionic skin and reported tough hydrogels and elastomers with fatigue resistance
**Suppl. Table 3.** Comparison of the stretchability, self-healability, and strain-stiffening properties among typical fiber mesh/fabric-reinforced hydrogels and elastomers
**Suppl. Table 4.** Comparison of overall sensing-related performance between hybrid ionic skin and reported intrinsically stretchable ionic conductors
**Suppl. Table 5.** Final results of the multiplication of the signs of each cross-peak in 2DCOS

**References S1-S69**

**Suppl. Movie 1.** Strain-stiffening demonstration of hybrid ionic skin
**Suppl. Movie 2.** Single-edge notch tension test of hybrid ionic skin
**Suppl. Movie 3.** Self-healing of hybrid ionic skin

## Extended Methods

**Preparation of PAA/EMI ES ionogel and hybrid ionogel.** The ionogel was prepared by mixing PAA solution (35 wt% in $H_2O$) and EMI ES with a mass ratio of 2:1. After degassing, the solution was poured into a polytetrafluoroethylene mold and ionogel formed in a constant temperature & humidity chamber (RH 60%, 25 °C) for 24 h. The synthesis process of hybrid ionogel was the same as that of hybrid ionic skin.

**Preparation of PAA/PDMAEA-Q ionoelastomer and hybrid ionoelastomer.** The precursor solution was prepared by mixing a cationic monomer DMAEA-Q and PAA (35 wt% in $H_2O$) with a mass ratio of 4:1 and 2-oxoglutaric acid (0.1 mol % with respect to DMAEA-Q) as the photoinitiator. The solution was polymerized by UV light at 365 nm for 1 h in a glass mold consisting of two parallel glass slides. The synthesis process of hybrid ionoelastomer was the same as that of hybrid ionic skin.

**Two-dimensional correlation spectroscopy (2DCOS).**

2DCOS is a mathematical method proposed by Noda, which is very suitable for tracking and analyzing the event sequence of spectral variations under various external disturbances, such as humidity, temperature, time, pressure, and other physical variables[S1,S2]. By spreading the original 1D spectra in the second dimension, the spectral resolution can be effectively enhanced, allowing the extraction of hidden subtle information about molecular motions or conformational changes in addition to traditional analysis. 2DCOS generally consists of two kinds of correlation maps: one is synchronous spectrum reflecting the simultaneous change of two wavenumbers, and the other is asynchronous spectrum which can effectively improve spectral resolution. In the synchronous spectrum, there are auto-peaks and cross-peaks along and off the diagonal respectively, while in the asynchronous spectrum, there are only cross-peaks. According to Noda's rule, the sequence can be judged as follows: if the multiplication of the signs of cross-peaks ($v_1$, $v_2$, and assume $v_1 > v_2$) in synchronous and asynchronous spectra is positive, the change at $v_1$ occurs prior to or earlier than $v_2$, and vice versa[S3]. For convenience, in Supplementary Table 5, the same sign in synchronous and asynchronous spectra is represented by "+", otherwise it is represented by "-".

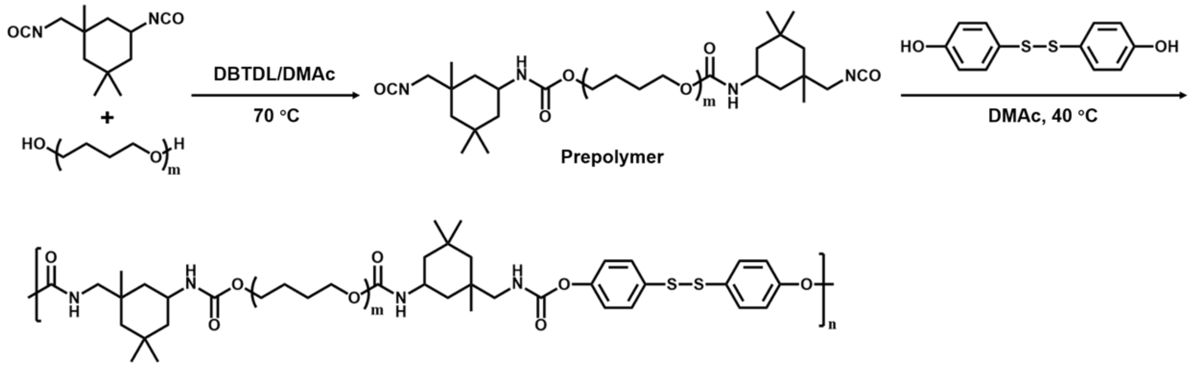


**Supplementary Fig. 1.** Synthesis of self-healing PU.

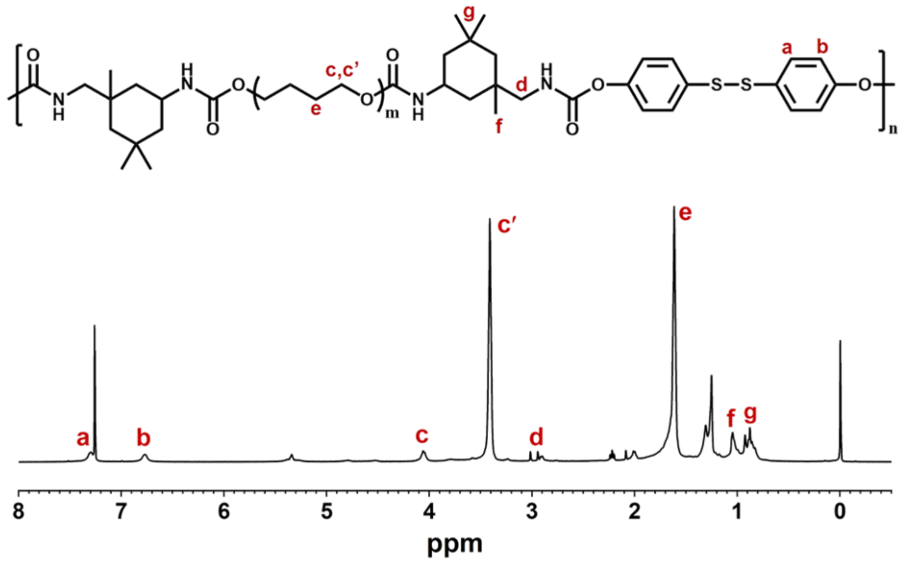


**Supplementary Fig. 2.** $^{1}$H NMR spectrum of self-healing PU**.** The used solvent is $CDCl_3$.

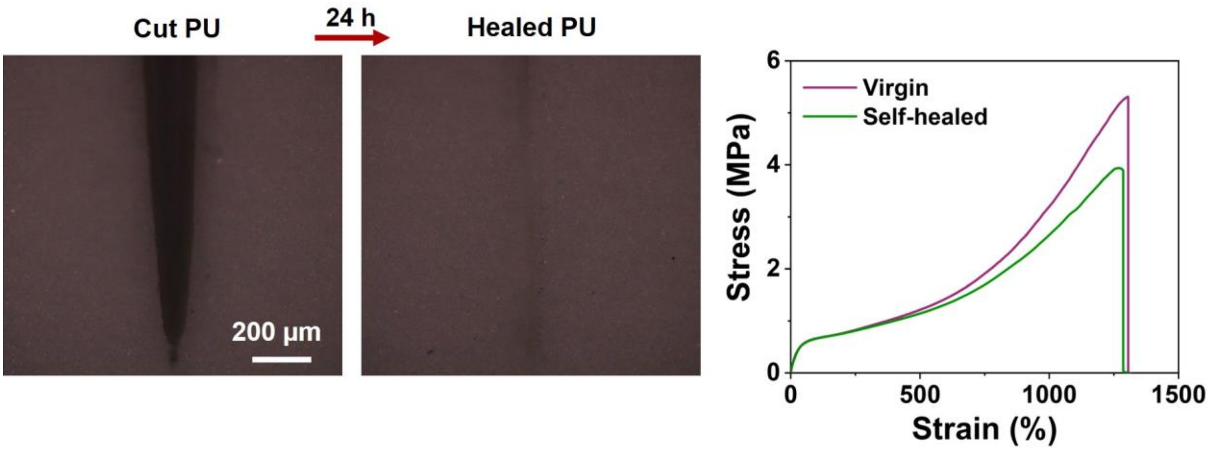


**Supplementary Fig. 3.** Optical micrographs and tensile stress-strain curves of a precut PU film before and after healing for 24 h at 25 °C. The Young′s modulus of PU film was calculated to be 2.8 MPa.

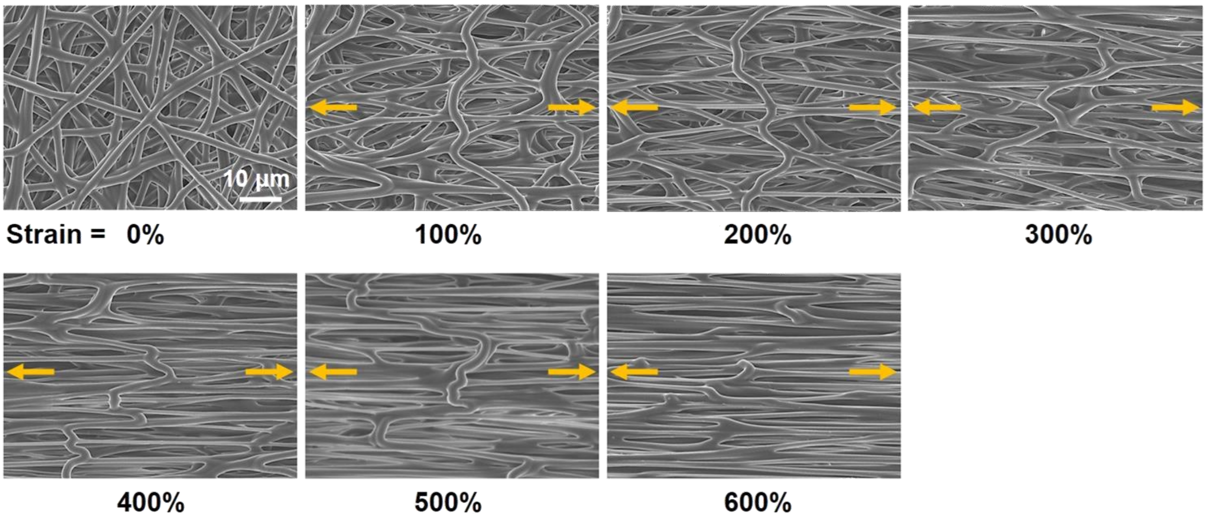


**Supplementary Fig. 4.** SEM images of self-healing PU nanomesh under different strains. The nanofibers are highly aligned in the stretched direction (indicated by yellow arrows).

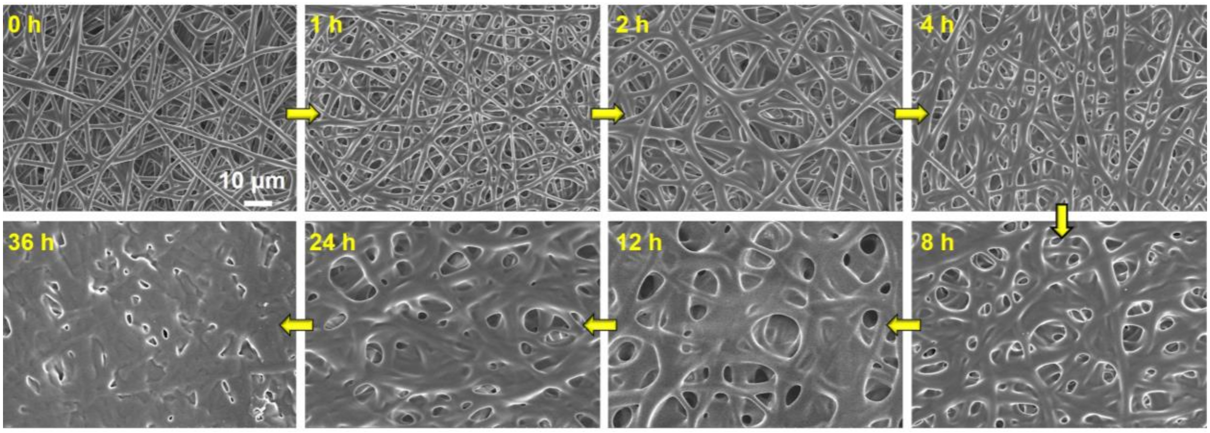


**Supplementary Fig. 5.** SEM images showed the time-dependent self-fusion process of self-healing PU nanomesh at 25 °C.

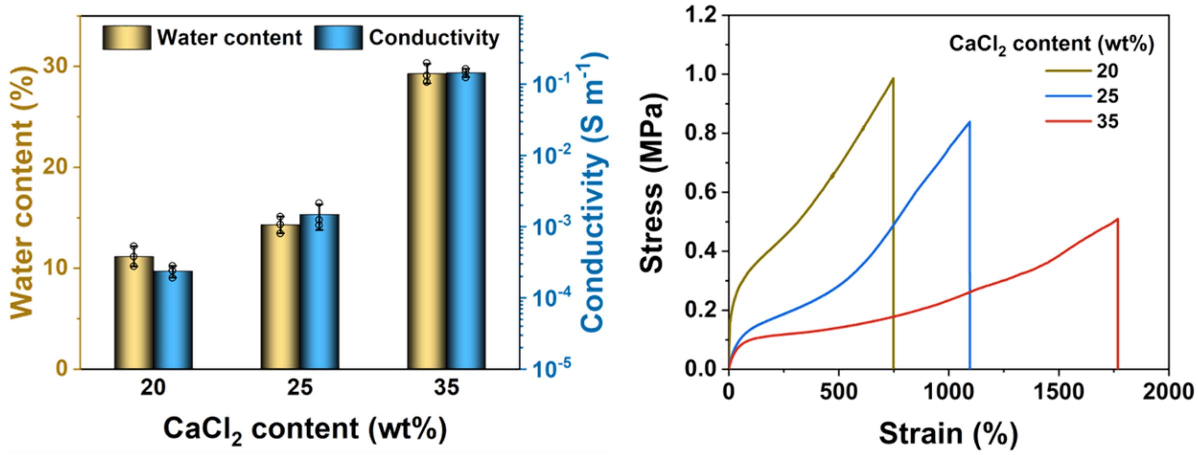


**Supplementary Fig. 6.** Water contents, ionic conductivities, and stress-strain curves of P(AAm-*co*-AA)/$CaCl_2$ ionic matrices with different $CaCl_2$ contents. The $CaCl_2$ content is referred to the weight proportion in the dry samples. Otherwise stated, all the samples mentioned hereafter have reached moisture equilibrium under RH 60%, 25 °C. Data are presented as mean values ± SD, n = 3 independent samples.

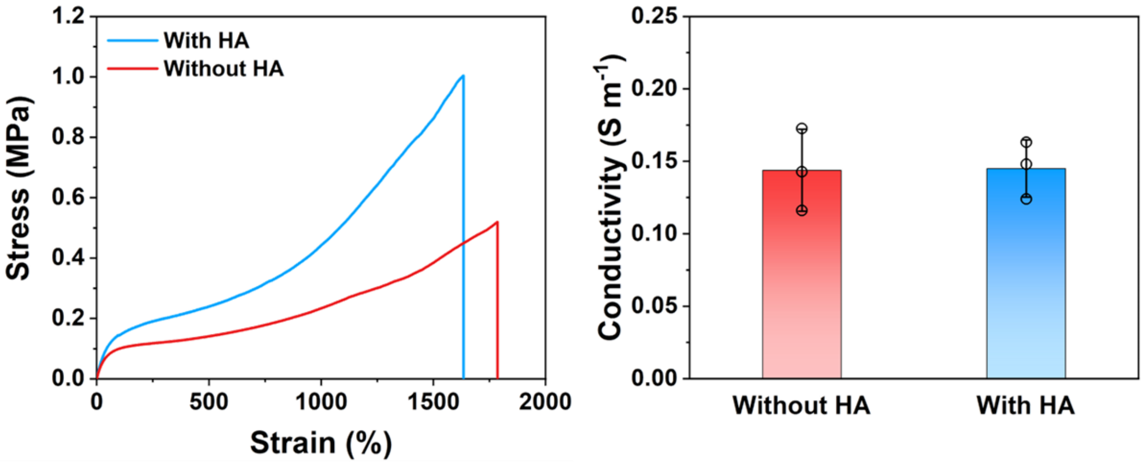


**Supplementary Fig. 7.** Stress-strain curves and ionic conductivities of P(AAm-*co*-AA)/$CaCl_2$ ionic matrices with/without HA. The added content of HA is 1 wt% with respect to P(AAm-*co*-AA). The $CaCl_2$ content is kept at 35 wt%. The addition of HA increased the tensile strength of P(AAm-*co*-AA)/$CaCl_2$ ionic matrix without sacrificing stretchability and ionic conductivity. Adding HA more than 1 wt% would significantly increase the matrix's modulus and reduce the stretchability. Conductivity data are presented as mean values ± SD, n = 3 independent samples.

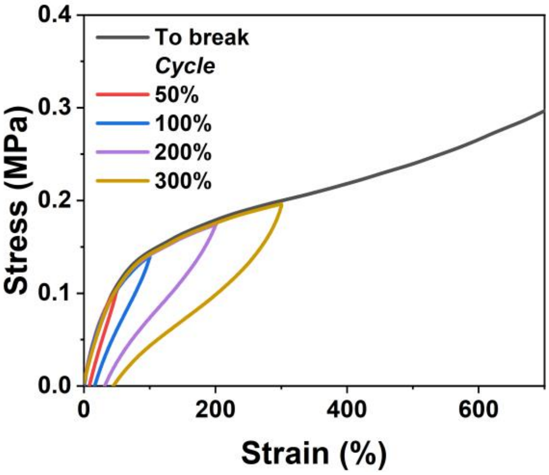


**Supplementary Fig. 8.** Cyclic stress-strain curves of P(AAm-*co*-AA)/HA/$CaCl_2$ ionic matrix at increasing strains. The strain rate was fixed to 3.3% $s^{-1}$, and waiting time was 15 min. In the presence of HA, the ionic matrix was highly elastic with almost identical loading curves to the single tensile curve to break. Obvious hysteresis loops are observed in the loading-unloading cycles, suggesting large energy dissipation through dynamic interchain crosslinks.

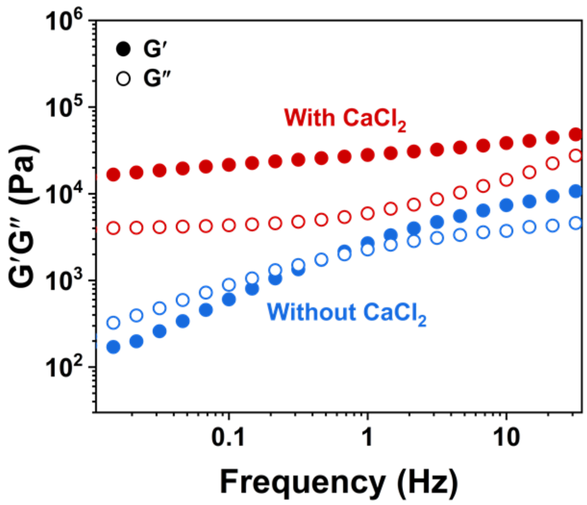


**Supplementary Fig. 9.** Frequency dependencies of the storage (G′) and loss (G″) moduli of the P(AAm-*co*-AA)/HA aqueous solutions with/without $CaCl_2$. The concentrations of polymer and added $CaCl_2$ are 45.5 wt% and 24.5 wt%, respectively.

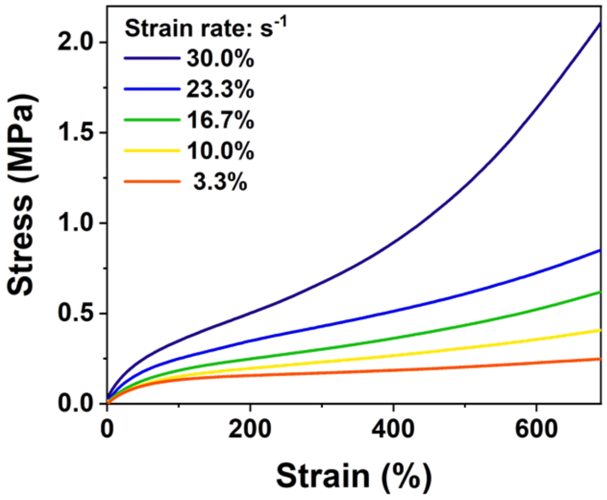


**Supplementary Fig. 10.** Strain rate-dependent tensile curves of P(AAm-*co*-AA)/HA/$CaCl_2$ ionic matrix. The strain rate dependence reveals the supramolecular nature of ionic matrix.

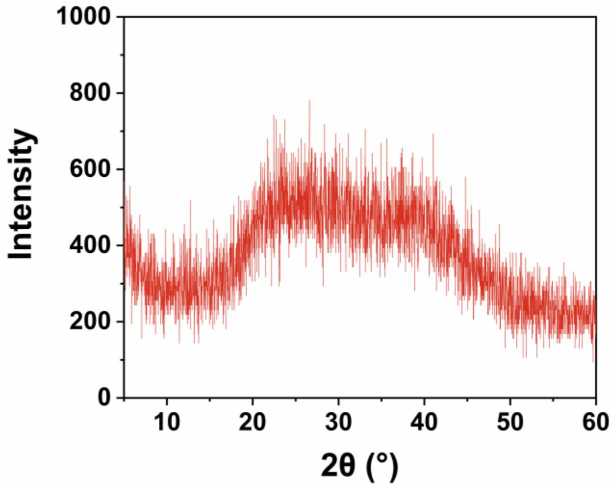


**Supplementary Fig. 11.** X-ray diffraction (XRD) profile of P(AAm-*co*-AA)/HA/$CaCl_2$ ionic matrix. There are no observed $CaCl_2$ crystallites, suggesting that $CaCl_2$ is fully dissolved in the polymer matrix.

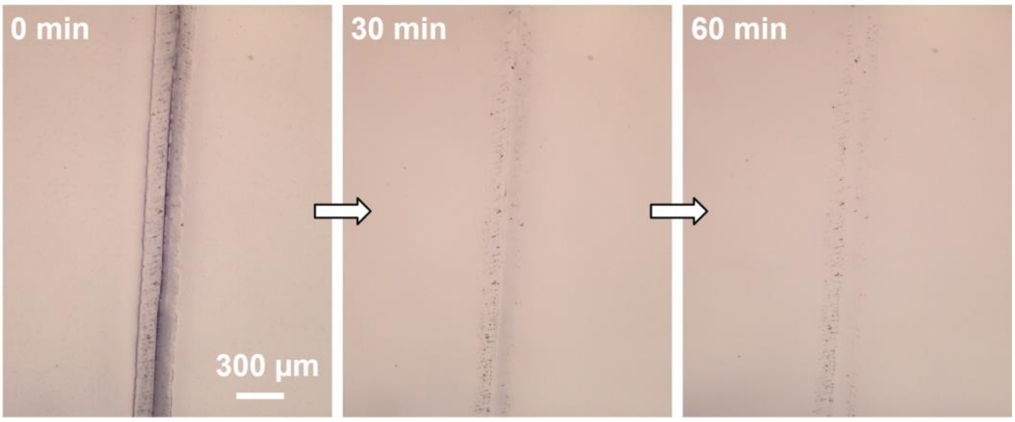


**Supplementary Fig. 12.** Optical microscopic images of a precut ionic matrix upon healing at RH 80% for 1 h.

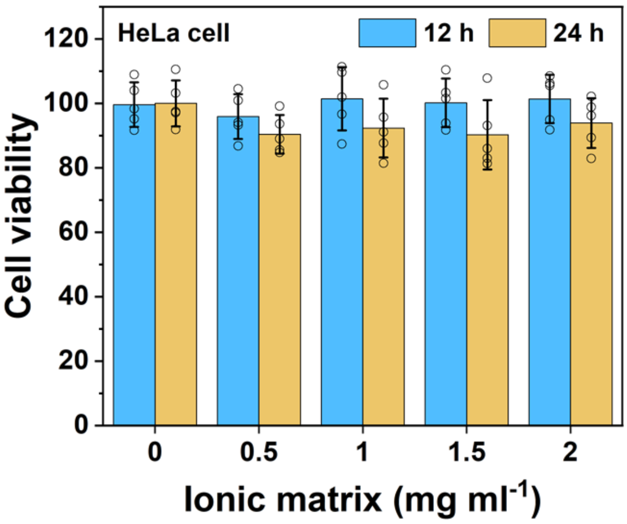


**Supplementary Fig. 13.** Cell viability test of HeLa cells incubated with different amounts of ionic matrix for 12 h and 24 h, respectively. The ionic matrix is proved to have very good biocompatibility (cell viability >90%). Data of cell viability are expressed as mean values ± SD, n = 5 independent solutions.

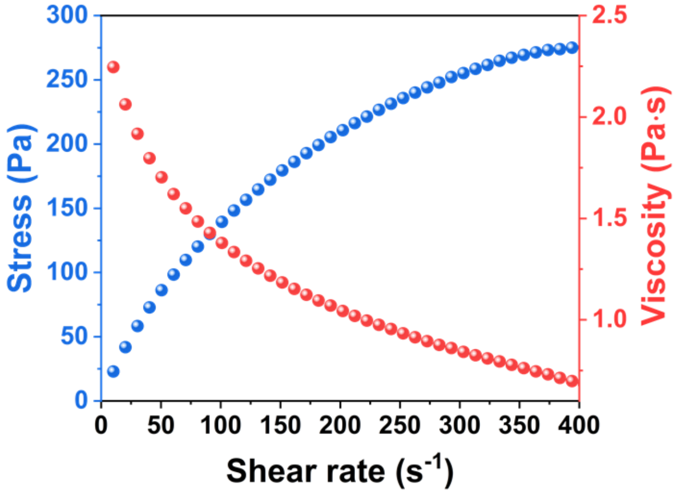


**Supplementary Fig. 14.** Shear-thinning behavior of the ionic matrix precursor. The precursor has a total polymer concentration of 10.8 wt% and $CaCl_2$ concentration of 5.7 wt%.

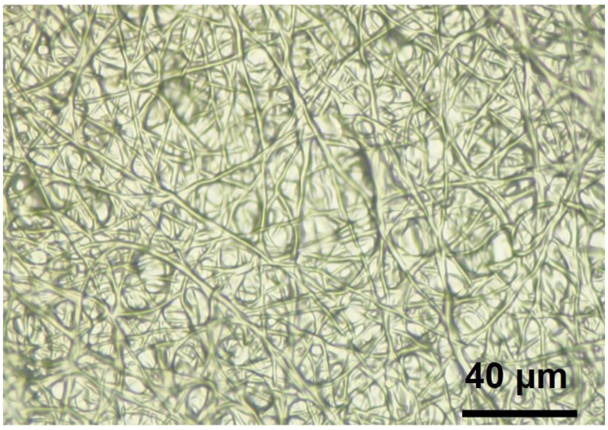


**Supplementary Fig. 15.** Optical microscopic image of hybrid ionic skin. It is observed that PU nanofibers are randomly distributed in the ionic matrix.

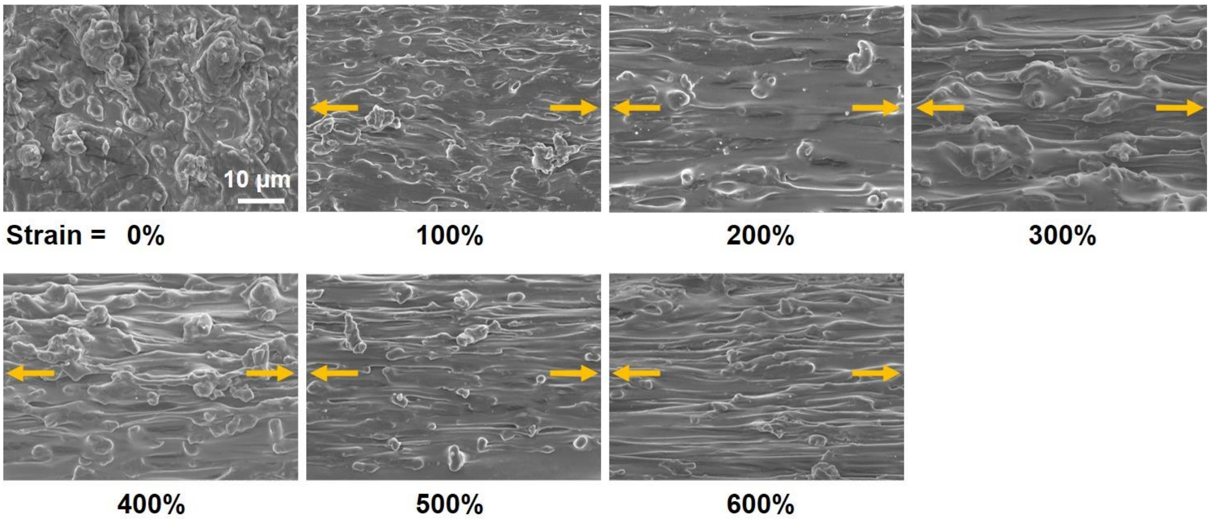


**Supplementary Fig. 16.** Cross-sectional SEM images of hybrid ionic skin under different strains. Yellow arrows indicate the stretching directions.

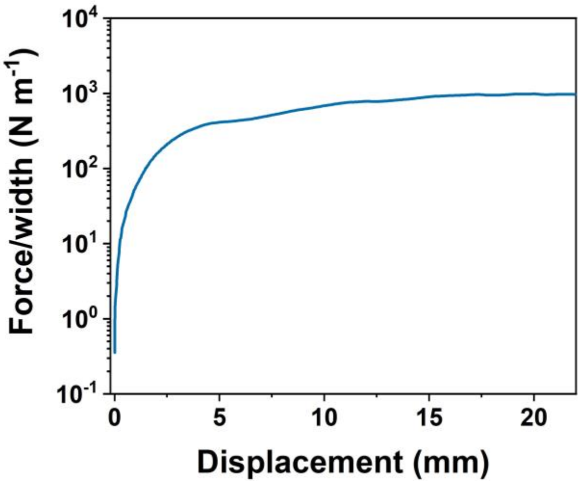


**Supplementary Fig. 17.** 90$^{o}$ peeling test for measuring the adhesion strength between ionic matrix and PU nanomesh. The strong fiber-matrix adhesion prevents debonding and sliding, reaching a very high interfacial toughness of ~820 J m$^{-2}$.

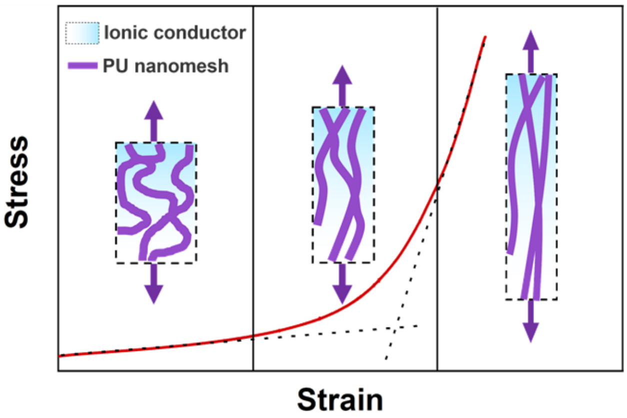


**Supplementary Fig. 18.** Schematic nonlinear elasticity of hybrid ionic skin. The strain-stiffening response is mainly due to the orientation and gradual load-bearing of PU nanofibers[S4].

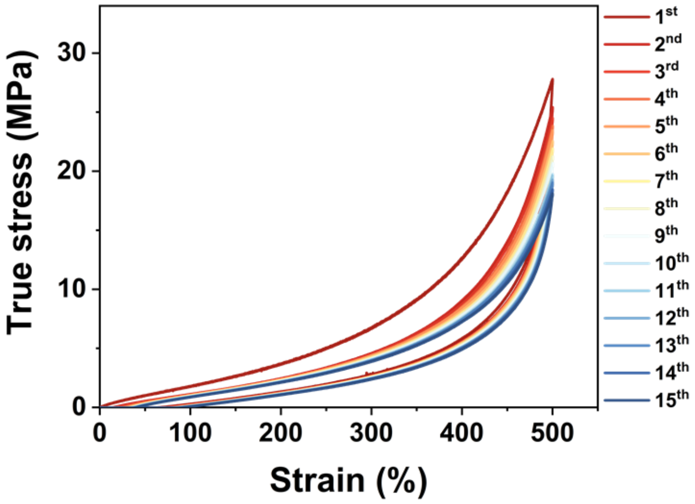


**Supplementary Fig. 19.** Cyclic tensile stress-strain curves of hybrid ionic skin at the fixed strain of 500%. Waiting time was set to 5 min.

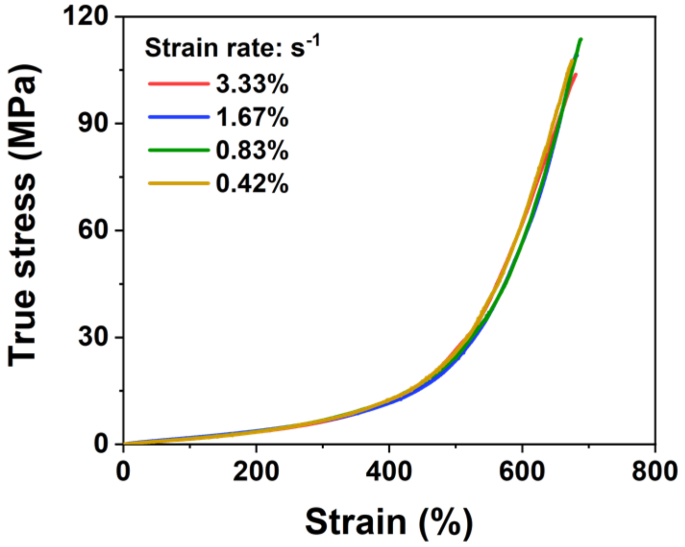


**Supplementary Fig. 20.** Tensile stress-strain curves of hybrid ionic skin at different strain rates.

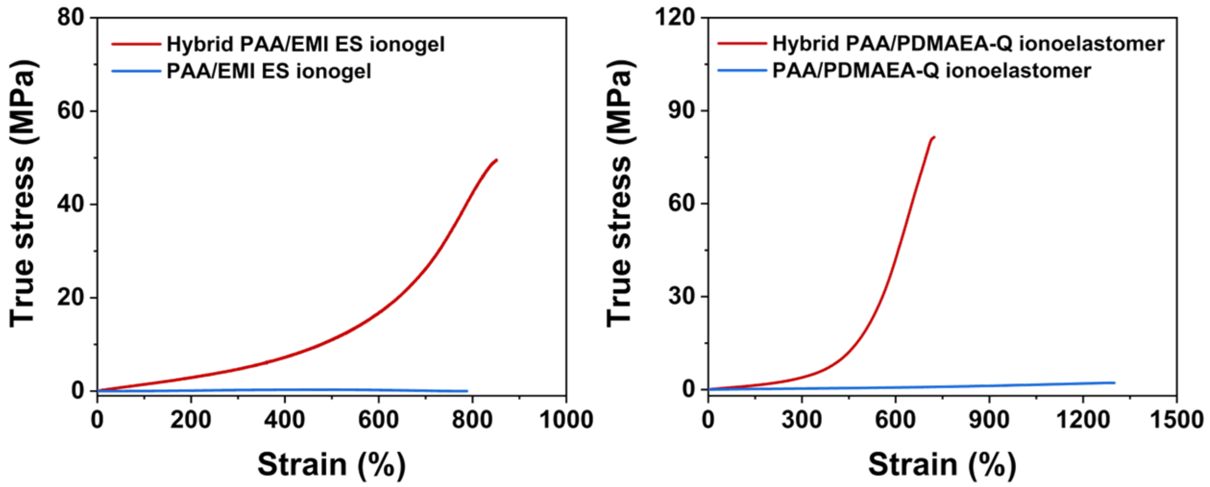


**Supplementary Fig. 21.** Stress-strain curves of PAA/EMI ES ionogel and PAA/PDMAEA-Q ionoelastomer as well as their nanomesh hybrid counterparts. After hybridizing with PU nanomesh, apparent J-shaped strain-stiffening behavior can be realized.

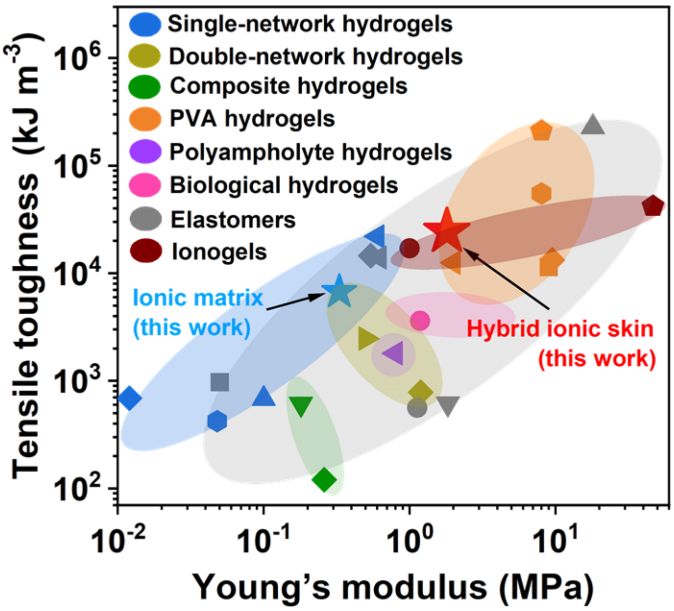


**Supplementary Fig. 22.** Comparison of the tensile toughness and Young′s modulus of hybrid ionic skin with previous tough hydrogels and elastomers. Detailed data is presented in Supplementary Table 2.

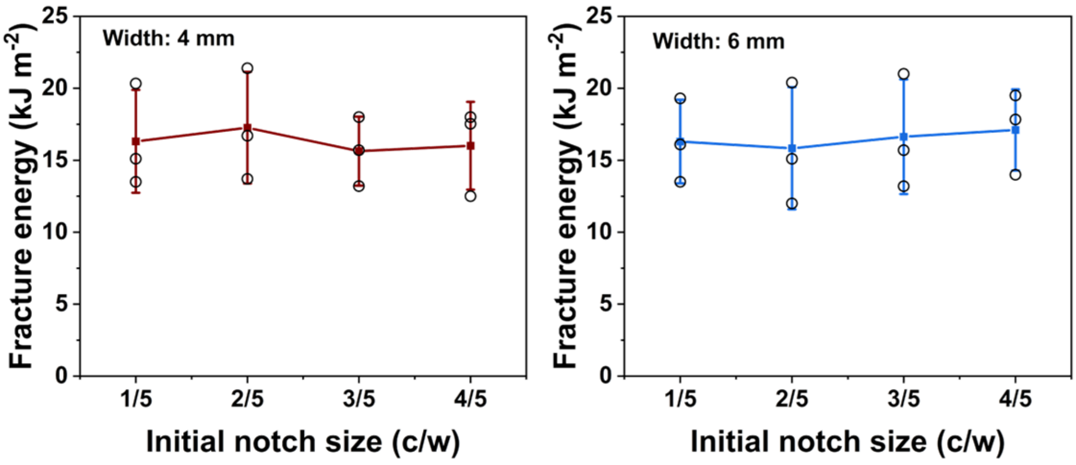


**Supplementary Fig. 23.** Fracture energies of hybrid ionic skin obtained from single-edge notch tension (SENT) tests with different initial notch sizes and sample widths. c is the notch size and d the sample width. The calculated fracture energy is almost independent of the initial notch size and sample width. Data are presented as mean values ± SD, n = 3 independent hybrid ionic skins.

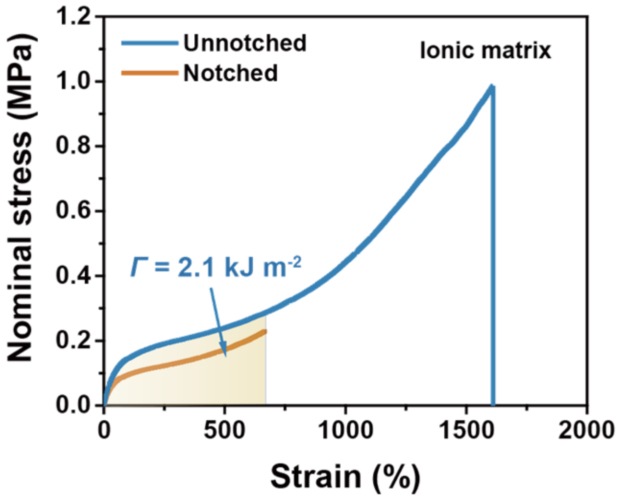


**Supplementary Fig. 24.** Nominal stress-strain curves of unnotched and notched ionic matrices. According to the single-edge notch tension test, the calculated fracture energy of neat ionic matrix is 2.1 kJ m$^{-2}$.

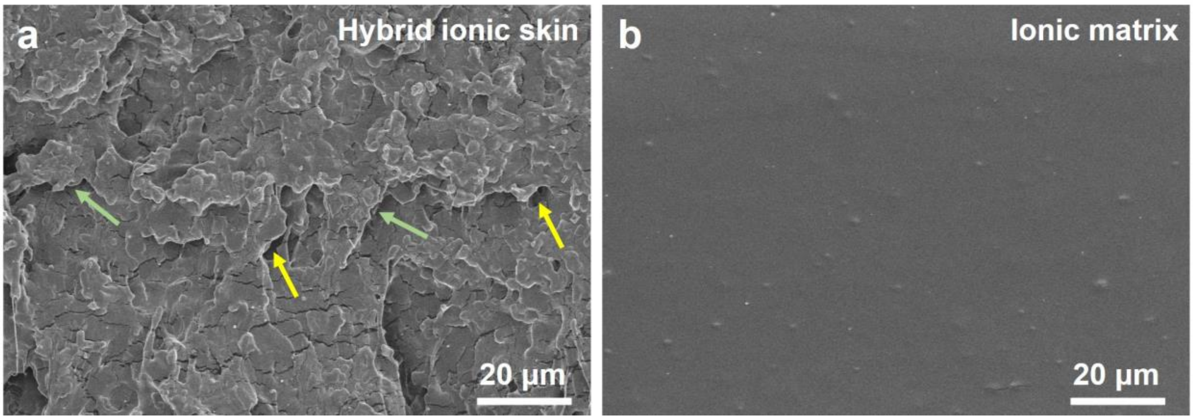


**Supplementary Fig. 25.** **a** SEM image of the fractured surface of notched hybrid ionic skin, which reveals a typical fiber-reinforced toughening mechanism from the signs of fiber pulling-out (yellow arrows) and crack deflection (green arrows). Therefore, as stretched, the PU nanofibers can effectively blunt cracks from propagation by creating a number of force transfer lengths. **b** As a contrast, the notched ionic matrix showed very smooth fractured surface after tensile test, indicating a fast crack propagation behavior.

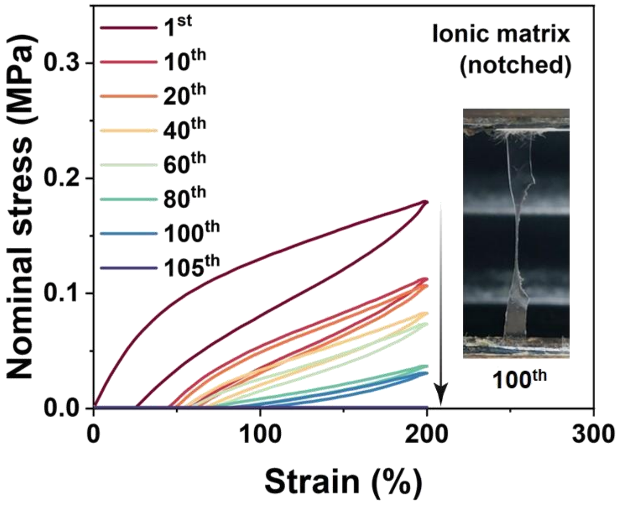


**Supplementary Fig. 26.** Cyclic tensile curves of notched ionic matrix at 200% strain. The inset is the stretched sample at the 100th cycle which was almost fractured.

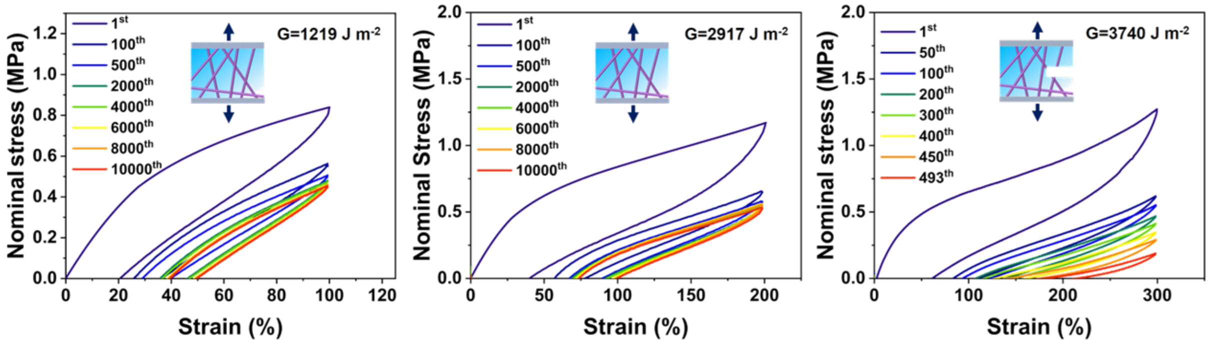


**Supplementary Fig. 27.** Stress decays of hybrid ionic skin at different energy release rates ($G$) in the fatigue fracture tests. When strain was smaller than 200% (G ≤ 2,917 J m$^{-2}$), the notch was always in the passivated state, and the notched sample could withstand 10,000 cycles' stretching without crack propagation. However, when strain was 300% (G = 3,740 J m$^{-2}$), matrix rupture would occur.

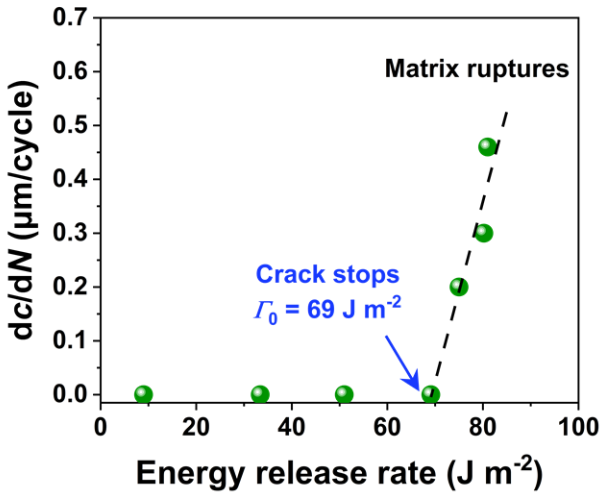


**Supplementary Fig. 28.** Crack extension per loading cycle, d$c$/d$N$, versus energy release rate for ionic matrix.

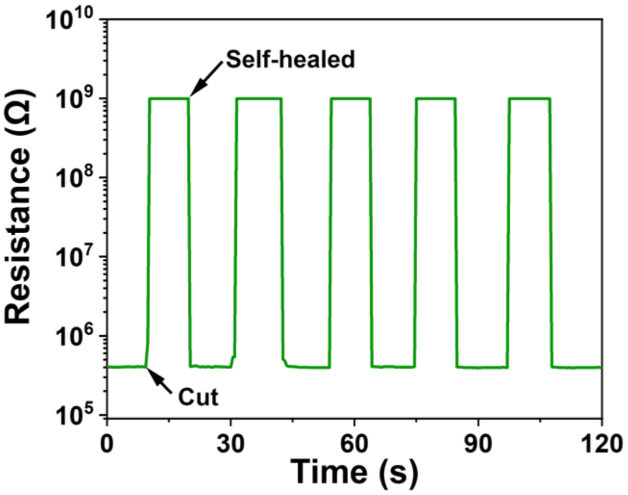


**Supplementary Fig. 29.** Healed ionic conductivity of hybrid ionic skin as monitored by real-time resistance measurement.

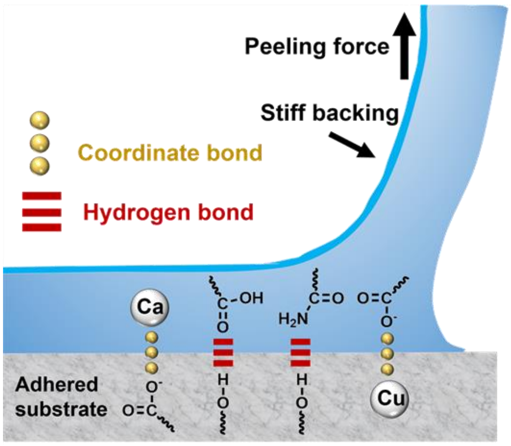


**Supplementary Fig. 30.** Schematic illustration of 90° peeling test for bonding strength and possible adhesion mechanism between hybrid ionic skin and various substrates. A stiff backing was introduced to prevent elongation of hybrid ionic skin along the peeling direction.

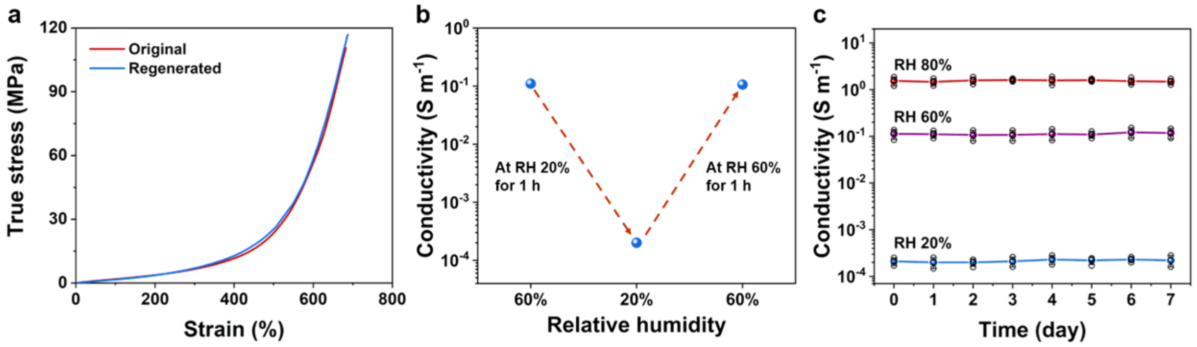


**Supplementary Fig. 31. a,b** Tensile curves and ionic conductivities of original and regenerated hybrid ionic skins. The regeneration process was performed by drying the sample at RH 20% and making it re-absorb moisture at RH 60% for 1 h. **c** Ionic conductivities of hybrid ionic skin within one week under different humidities at 25 °C. Data are presented as mean values ± SD, n = 3 independent hybrid ionic skins.

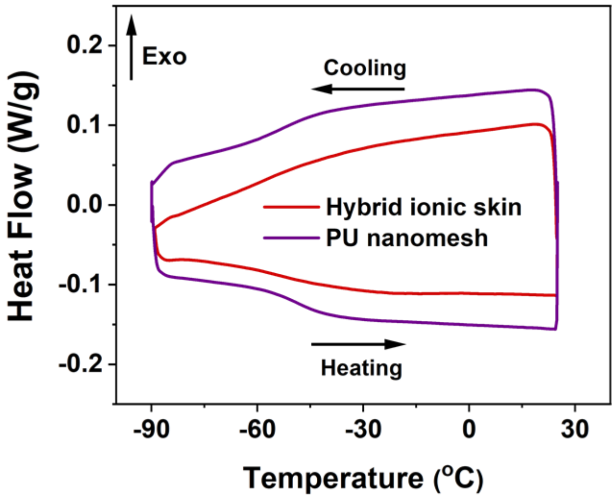


**Supplementary Fig. 32.** DSC heating and cooling curves of PU nanomesh and hybrid ionic skin equilibrated at RH 60%, 25°C. The hybrid ionic skin sample for DSC measurement contained about 30.5 wt% water, as evaluated by the weighing method after lyophilization. The glass transition temperature of PU nanomesh is about -42 °C, low enough for most of anti-freezing applications without the loss of fiber's flexibility.

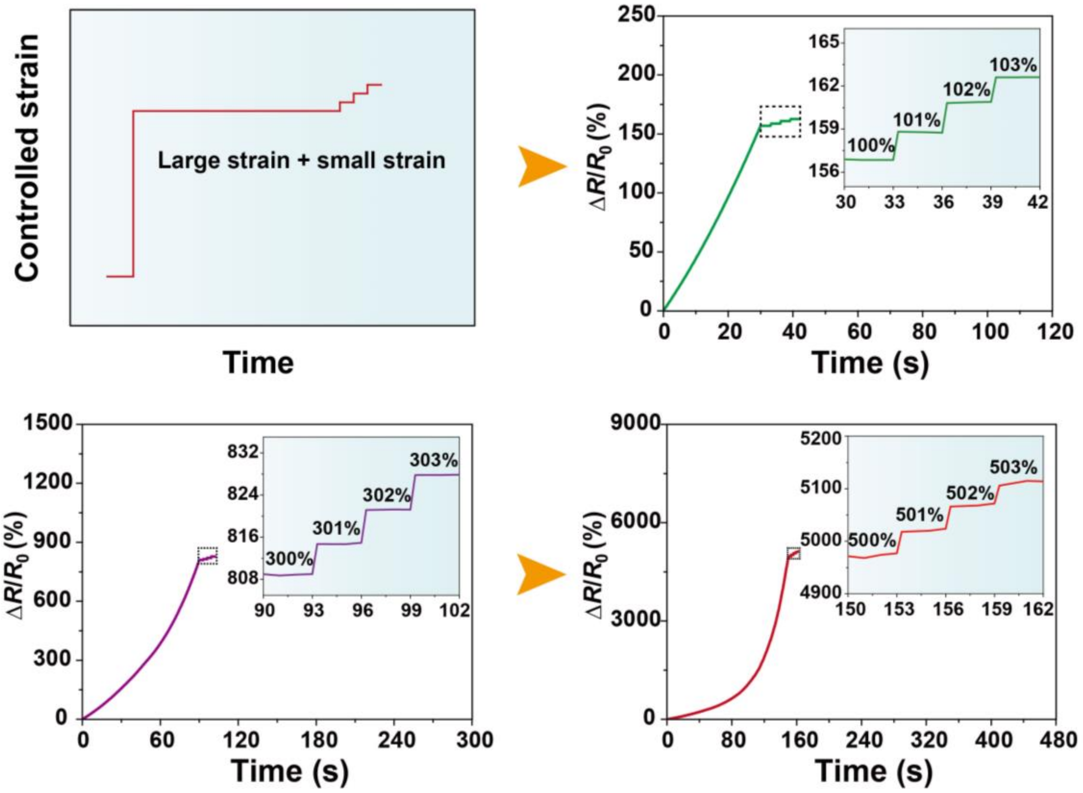


**Supplementary Fig. 33.** Resistance changes of hybrid ionic skin by superimposing gradient 1% strains on fixed strains (100%, 300%, 500%).

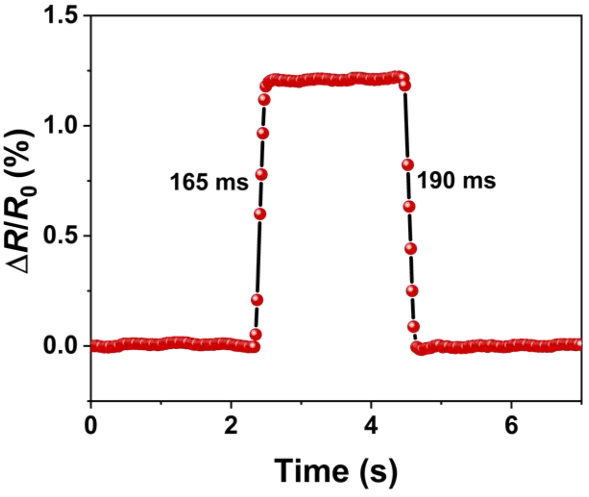


**Supplementary Fig. 34.** Response time of hybrid ionic skin sensor as stretched to and recovered from 1% strain.

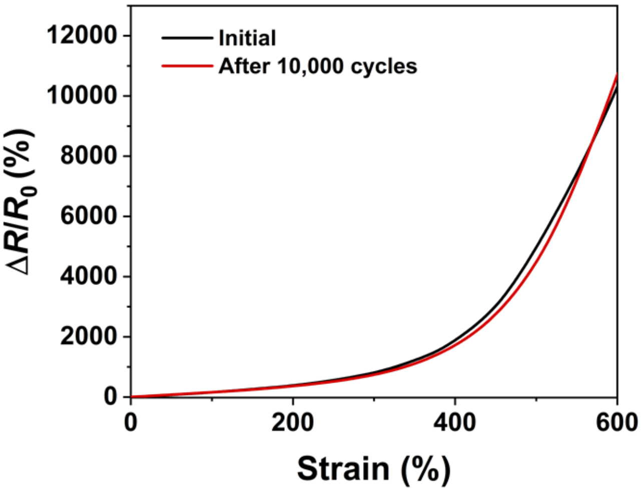


**Supplementary Fig. 35.** Strain-dependent resistance changes before and after stretching to 200% strain for 10,000 cycles.

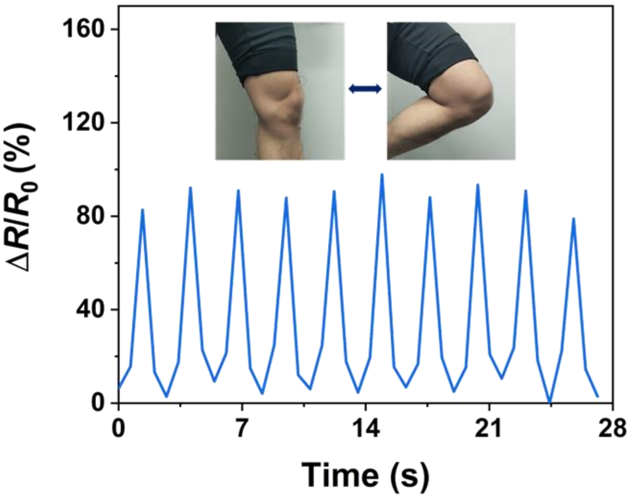


**Supplementary Fig. 36.** Monitoring knee bending with hybrid ionic skin sensor.

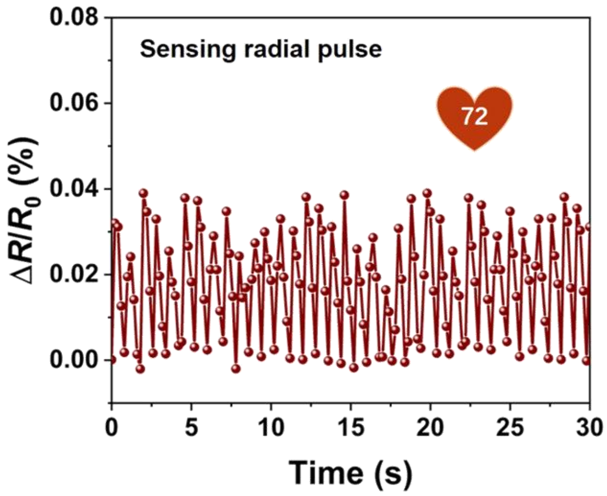


**Supplementary Fig. 37.** Monitoring human radial pulse by hybrid ionic skin attached on the wrist. By analyzing the output signal, the average radial pulse during relaxation was ca. 72 beats per minute.

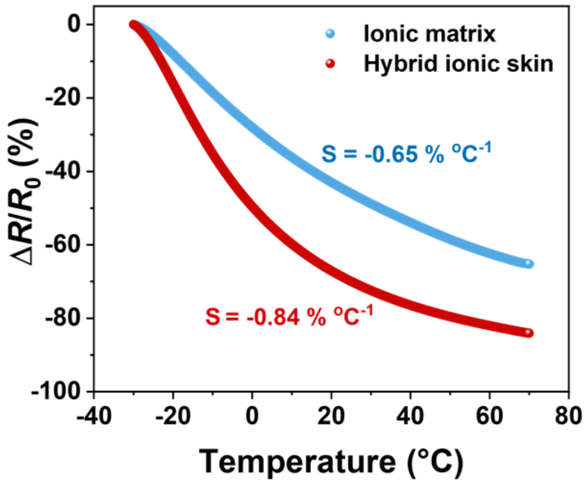


**Supplementary Fig. 38.** Temperature-dependent resistance changes of hybrid ionic skin and ionic matrix. The average thermal sensitivity of hybrid ionic skin is 0.84% $°C^{-1}$, much higher than neat ionic matrix (0.65% $°C^{-1}$). This may be due to the higher coefficient of thermal expansion (CTE) of PU nanofibers (170 ppm $°C^{-1}$) than ionic matrix (67 ppm $°C^{-1}$), which exerts additional internal compression to ion-conducting channels as temperature rises.

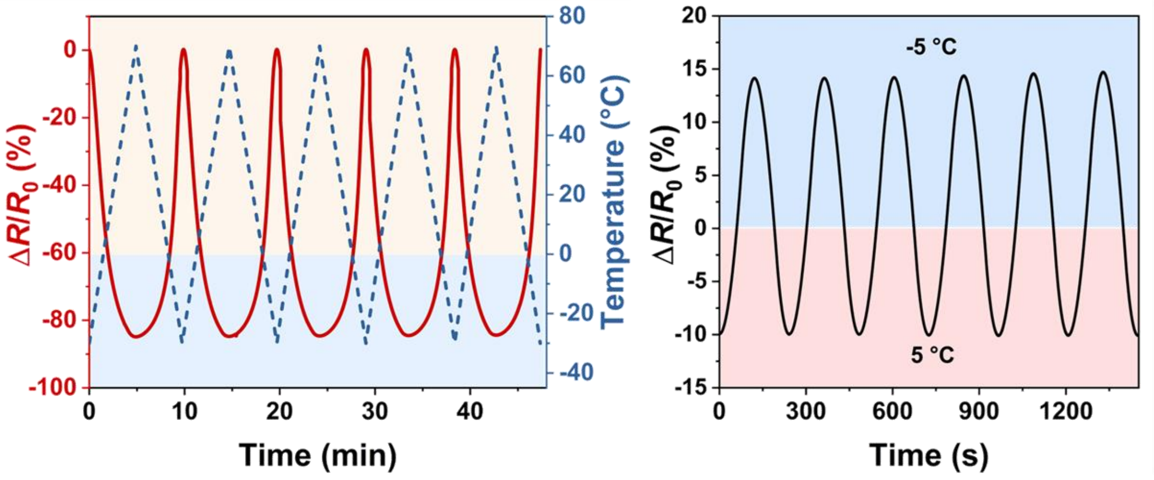


**Supplementary Fig. 39.** Temperature-dependent reversible resistance changes of hybrid ionic skin. The switching temperature ranges were set from -30 to 70 ℃ and from -5 to 5 ℃, respectively.

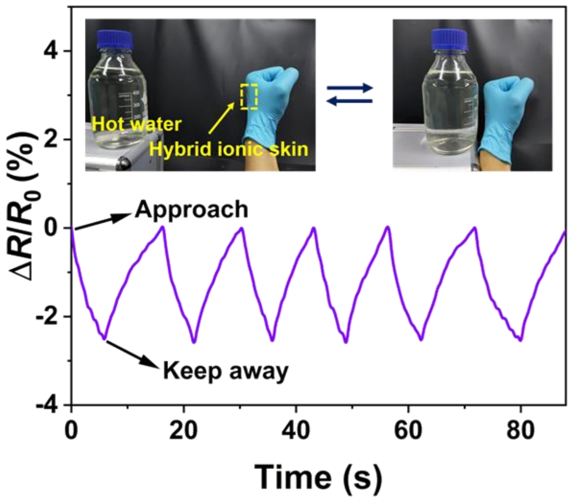


**Supplementary Fig. 40.** Real-time electrical response of the hybrid ionic skin adhered on human skin when a bottle of hot water (~90 °C) approached and moved away repeatedly.

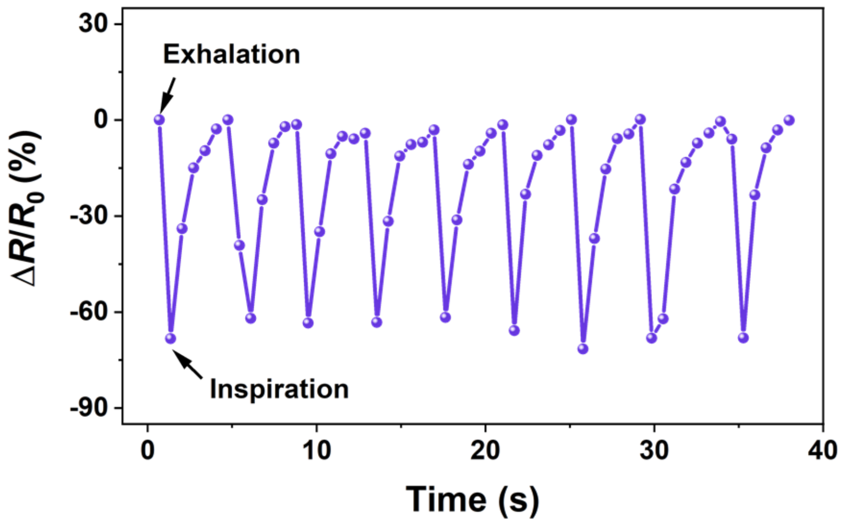


**Supplementary Fig. 41.** Monitoring human respiration by hybrid ionic skin. The breathing hybrid ionic skin immediately absorbed moisture from the exhaled high-humidity air, resulting in the instant increase of ionic conductivity, and thus reduced resistance. Conversely, when a person inhaled, dry air reached the sensor, generating a rising resistance.

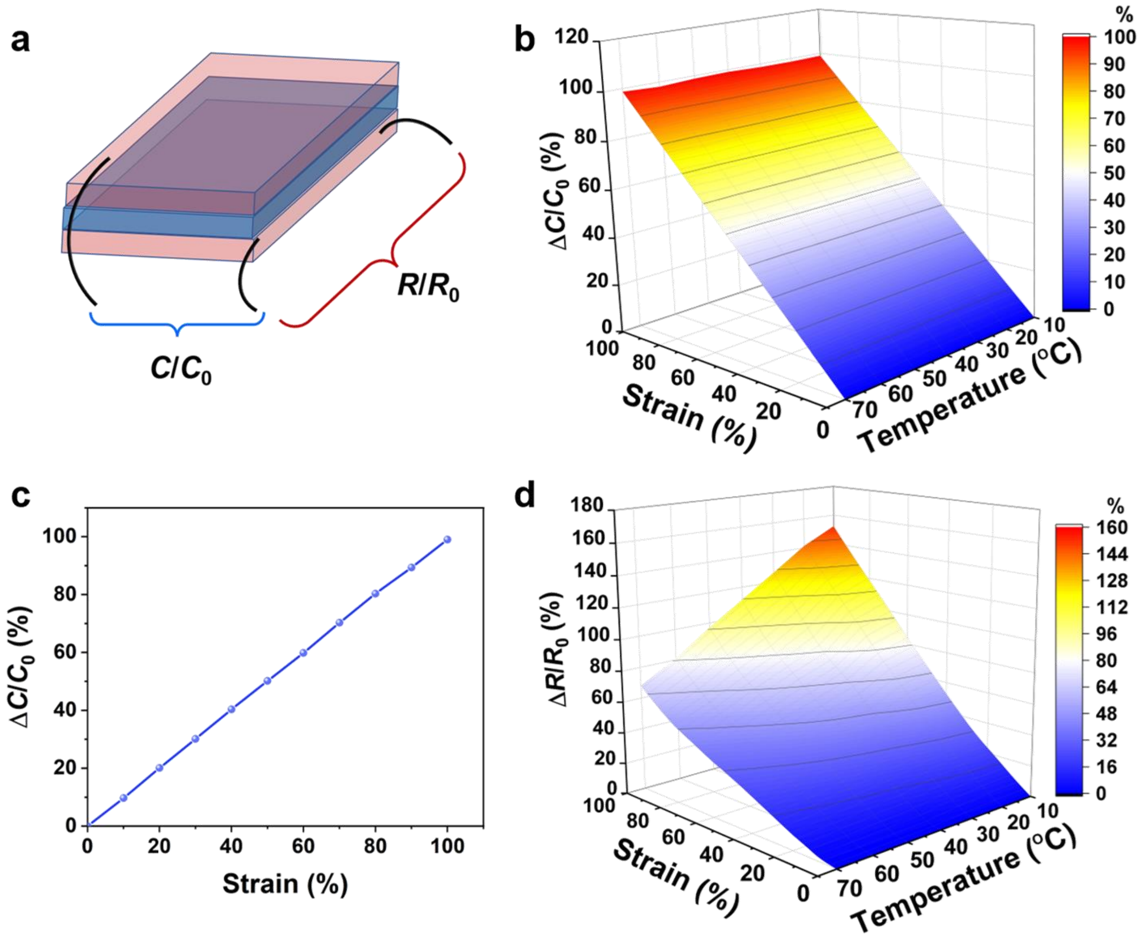


**Supplementary Fig. 42.** Decoupling the strain and temperature sensing by hybrid ionic skin-based capacitive sensor. **a** Schematic illustration of a multifunctional sensor (capacitance and resistance). The red layers and blue layer represent hybrid ionic skins and VHB elastomer, respectively. The sensor was encapsulated with VHB tape to avoid the influence of moisture on the sensing performance. **b** Strain- and temperature-dependent capacitive response. **c** Typical capacitance-strain curve of the sensor. **d** Strain- and temperature-dependent resistive response. It is clear that, the capacitance of the sensor is only sensitive to strain, yet the resistance is sensitive to both strain and temperature, which enables the decoupling of these two stimuli. At elevated temperatures, the strain sensitivities of resistance were significantly reduced. This is perhaps due to the promoted effect of strain-induced ionic conductivity increase, since at increasing temperatures the physical crosslinks in ionic matrix are weakened and thus network orientation becomes much easier.

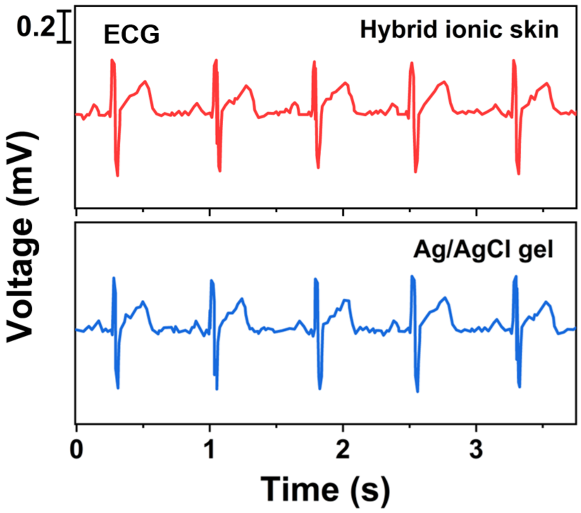


**Supplementary Fig. 43.** ECG signals of a volunteer recorded by hybrid ionic skin and commercial Ag/AgCl gel electrodes. The ECG waveforms obtained from the hybrid ionic skin electrode are almost identical to those obtained from the commercial gel electrode.

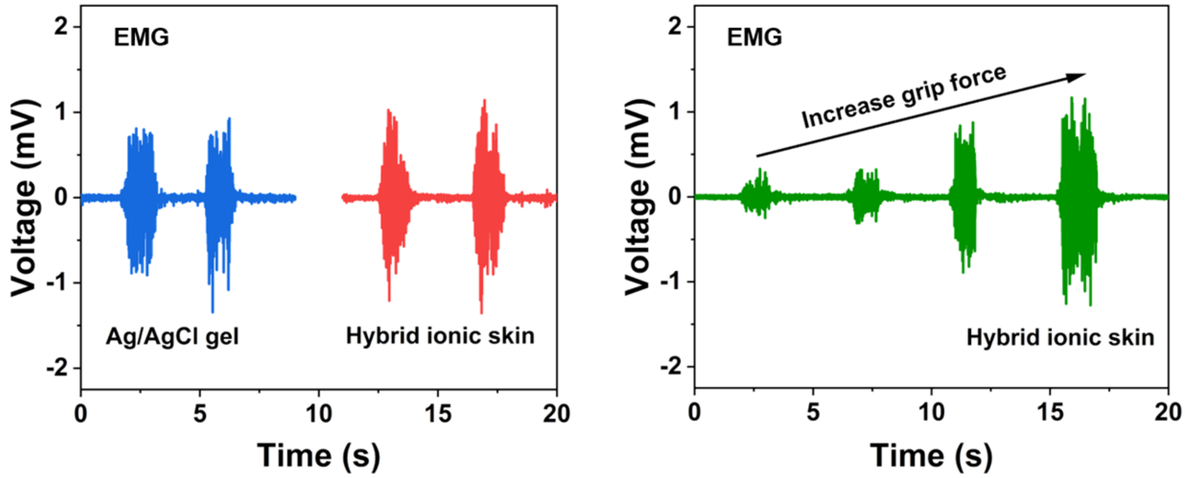


**Supplementary Fig. 44.** EMG signals of a volunteer recorded by hybrid ionic skin and commercial Ag/AgCl gel electrodes. For measurement, two pieces of hybrid ionic skins were discretely adhered on the inner forearm separated by a distance of 2 cm as a ground electrode and a measure electrode, respectively; another hybrid ionic skin as a reference electrode was placed at the elbow. The recorded EMG signals during repeated fist clenching and opening were reliable using the hybrid ionic skin electrode. Increasing the grip force induced larger EMG signals.

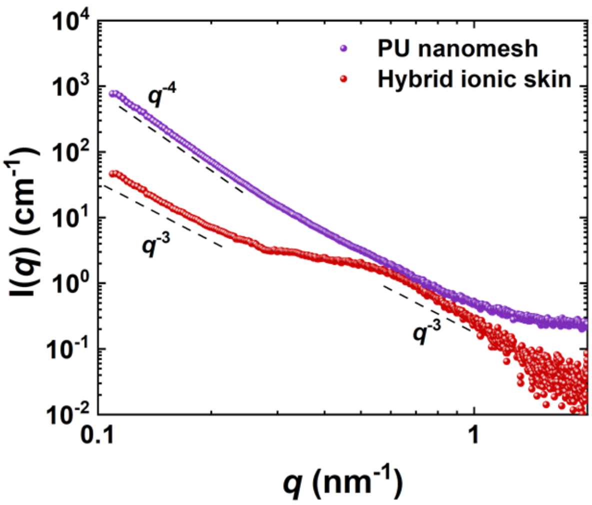


**Supplementary Fig. 45.** SAXS scattering intensity (I) vs scattering vector (q) profiles of hybrid ionic skin and PU nanomesh. The curves were integrated in the horizontal direction. Judging from the comparison, the plateau structure (corresponding to nanopores) appearing in the scattering curve of hybrid ionic skin above 0.3 $nm^{-1}$ mainly arises from ionic matrix. Moreover, it is also revealed that the unstretched PU nanomesh is composed of a dense fiber skeleton with large open pore structure ($q^{-4}$ scattering), and such a skeleton forms a hierarchical composite with ionic matrix.

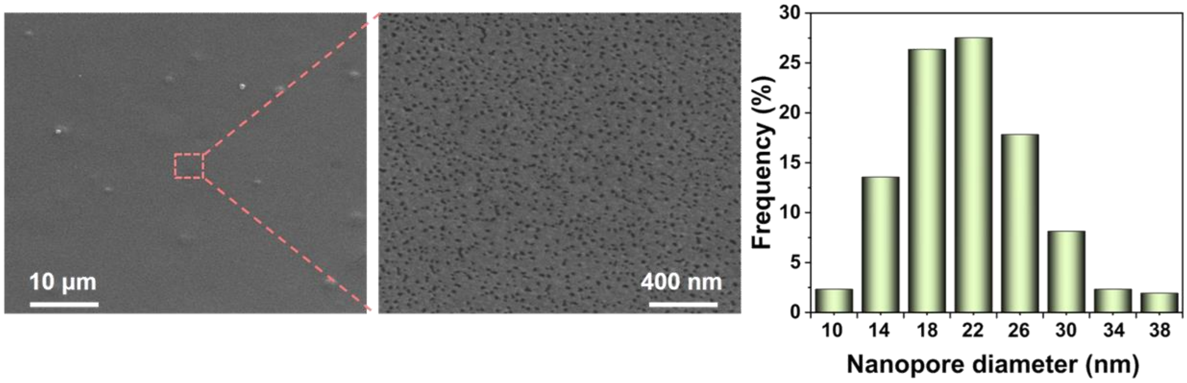


**Supplementary Fig. 46.** SEM images of vacuum-dried ionic matrix and statistical size distribution of nanopores. The mean size of nanopores is 21 nm.

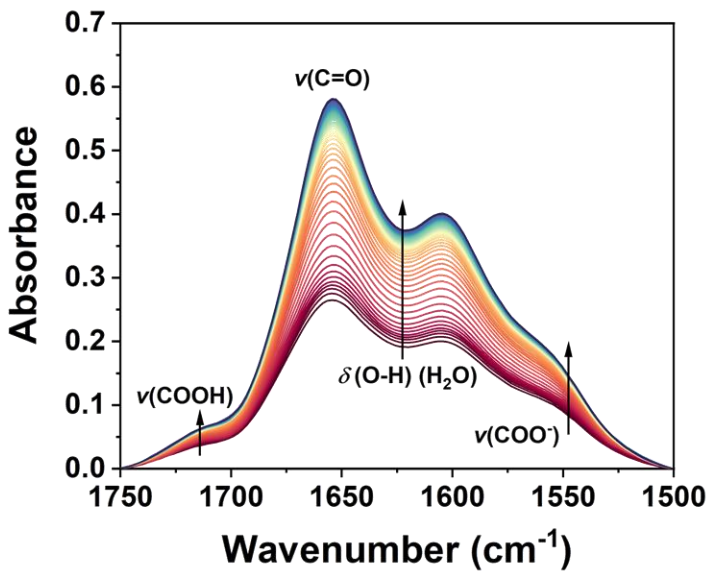


**Supplementary Fig. 47.** Time-resolved ATR-FTIR spectra of dried ionic matrix by absorbing moisture at RH 60%, 25 °C. The moisture-absorbing equilibrium finished in 50 min.

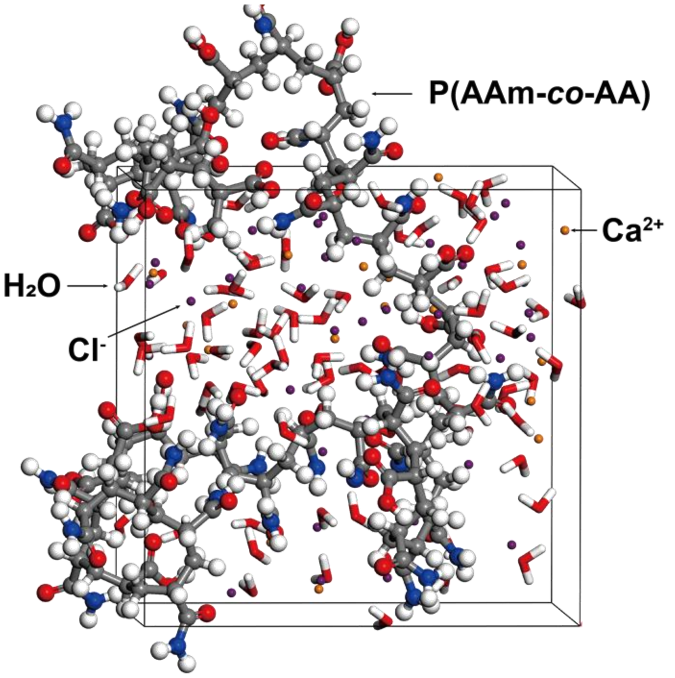


**Supplementary Fig. 48.** Amorphous cell of ionic matrix for molecular dynamics simulation. The cell contains one P(AAm-*co*-AA) chain with 40 repeating units, 20 $CaCl_2$, and 80 $H_2O$. The optimized cell shows that the polymer adopts a random-coil conformation with ions and water molecules homogenously distributed.

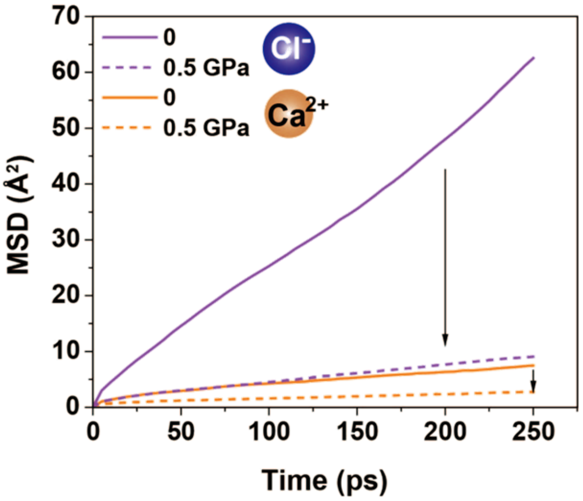


**Supplementary Fig. 49.** Computed mean square displacement (MSD) curves of $Ca^{2+}$ and $Cl^-$. $Cl^-$ is more mobile than $Ca^{2+}$ with a much higher diffusion coefficient, suggesting that $Cl^-$ is the main charge carrier in the ionic matrix. Applying a pressure of 0.5 GPa to the cell significantly reduced the ion diffusion coefficients of both $Ca^{2+}$ and $Cl^-$ (for $Ca^{2+}$, from $2.9\times10^{-6}$ to $1.1\times10^{-6}$ $cm^2$ $s^{-1}$; for $Cl^-$, from $24.8\times10^{-6}$ to $3.7\times10^{-6}$ $cm^2$ $s^{-1}$), corresponding to the drastic reduction of ionic conductivity.

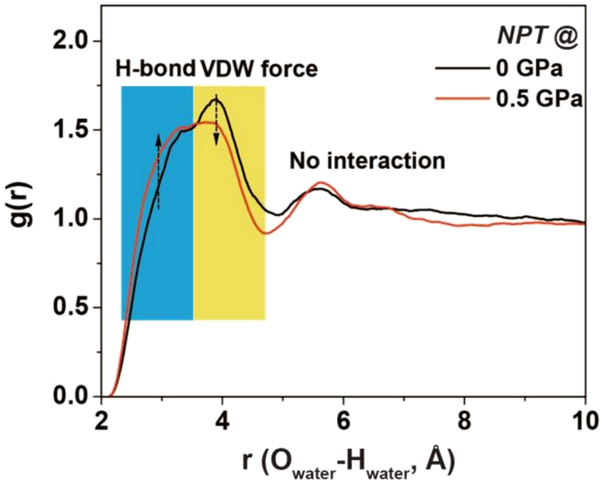


**Supplementary Fig. 50.** Radial distribution functions (RDFs) of O next to H atoms of water molecules under different pressures. Obviously, applying a conformational pressure induces the transformation from loosely associated water molecules to self-associated H-bonded ones, corresponding to water desorption from polymer chains.

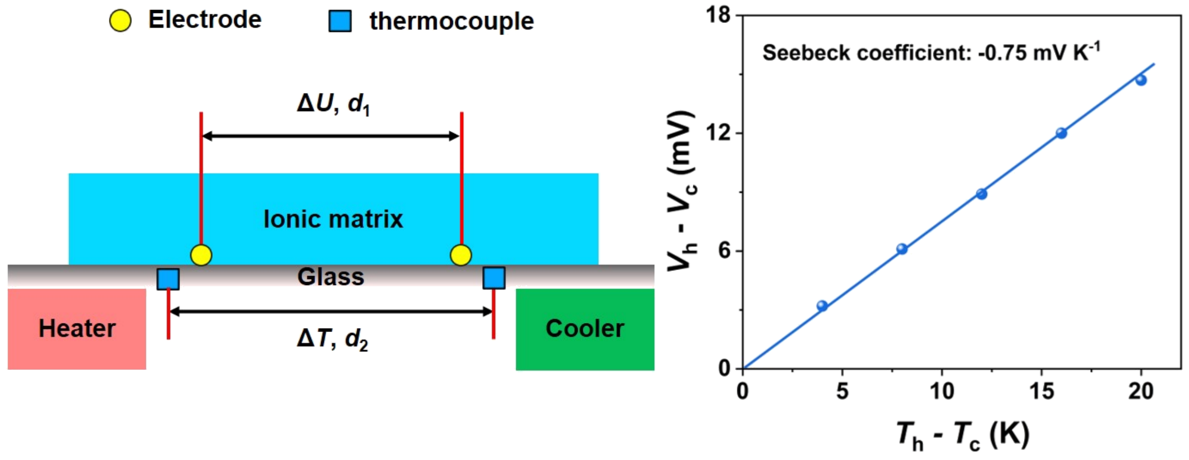


**Supplementary Fig. 51.** Open-circuit voltage variations of ionic matrix with temperature gradient measured by self-made temperature gradient platform. The open-circuit voltage between the two platinum electrodes with a diameter of 0.2 mm were evaluated with a multimeter (Keithley 2182A), and the temperature differences ($\Delta T$) between the two electrodes were measured by a multichannel temperature recorder (JK3000) equipped with *K*-type thermocouples. The thermoelectric Seebeck coefficient of ionic matrix was calculated as follows:

$$S_e = -\frac{V_h - V_c}{T_h - T_c}\frac{d_2}{d_1}$$

The measured negative Seebeck coefficient indicates a negative charge-dominating ion transportation behavior.

**Supplementary Table 1.** Comparison of the combinatory properties of human skin and hybrid ionic skin.

| Properties | Human skin | Hybrid ionic skin |
|---|---|---|
| **Stretchability (%)** | < 80 [S5] | 680 |
| **Initial modulus (MPa)** | 0.69-3.7 [S6,7] | 1.8 |
| **Final modulus (MPa)** | 54.0-83.3 [S6,7] | 67.5 |
| **Stiffness enhancement** | 15-70 | 37 |
| **Nominal strength (MPa)** | ~21.6 [S7] | 14 |
| **Toughness (MJ $m^{-3}$)** | ~3.6 [S6] | 24.4 |
| **Fracture energy ($\Gamma$, kJ $m^{-2}$)** | 1.7-2.6 [S8] | 16.3 |
| **Fatigue threshold ($\Gamma_0$, J $m^{-2}$)** | N/A | 2,950 |
| **Self-healing** | yes | yes |
| **Sensation** | Stretch, heat, touch, shear, moisture, vibration, pain, etc. | Stretch, heat, moisture |

Note: "N/A" indicates "not available" in the references. For skeleton muscle, $\Gamma_0$ ~1,000 J $m^{-2}$ [S9].

**Supplementary Table 2.** A rough comparison of mechanical performance among hybrid ionic skin and previously reported tough hydrogels and elastomers with fatigue resistance.

| Materials | | Self-healing | Young′s modulus (MPa) | Toughness (MJ $m^{-3}$) | Fracture energy (kJ $m^{-2}$) | Fatigue threshold (J $m^{-2}$) | Refs. |
|---|---|---|---|---|---|---|---|
| **Hybrid ionic skin** | | Yes | 1.8 | 24.4 | 16.3 | 2950 | This work |
| **P(AAm-co-AA)/HA/$CaCl_2$ ionic matrix** | | Yes | 0.3 | 6.7 | 2.1 | 69 | |
| **Composite hydrogels** | **Stiff skeleton-reinforced hydrogel** | No | ~0.18 | ~0.61 | ~4.6 | 441 | S10 |
| | **PDMS fiber-reinforced hydrogel** | No | 0.26 | ~0.12 | 4.1 | 1290 | S11 |
| | **TPN hydrogel** | No | ~0.45 | ~0.65 | 4.2 | N/A | S12 |
| **PVA hydrogels** | **Mechanical training PVA hydrogel** | No | 0.2 | ~2.0 | N/A | 1250 | S9 |
| | **FC-A PVA hydrogel** | No | 8 | ~55 | ~84.6 | 2740 | S13 |
| | **Dry-annealed PVA hydrogel** | No | ~9.5 | ~13.25 | 9 | 1000 | S14 |
| | **HA-PVA hydrogel** | No | ~8 | ~210 | 175 | 10500 | S15 |
| | **2D PVA/GO hydrogel** | No | ~9 | ~11.4 | N/A | 1500 | S16 |
| | **PVA-ferro/ferricyanide hydrogel** | No | ~2 | ~12.5 | 17.9 | 2500 | S17 |
| **Double-network hydrogels** | **PAAm/Ca-alginate hydrogel** | No | ~1.19 | ~0.781 | 3.4 | 35 | S18 |
| | **PAMPS/PAAM hydrogel** | No | ~0.5 | ~2.47 | 3.8 | 418 | S19 |
| **Single-network hydrogels** | **Highly entangled hydrogel** | No | ~0.1 | 0.68 | 1.46 | 200 | S20 |
| | **PAA-G8 hydrogel** | No | 0.012 | ~0.69 | ~0.9 | 126 | S21 |
| | **PAAm hydrogel** | No | ~0.048 | 0.42 | 0.6 | 64.5 | S22 |
| | **Slide-ring gel** | No | ~0.6 | 22 | 3.6 | N/A | S23 |
| **Elastomers** | **Ecoflex composite** | No | ~0.05 | ~0.97 | 5.7 | 500 | S24 |
| | **PDMS composite** | No | ~1.13 | 0.56 | ~10 | 160 | S25 |
| | **P(MEA-*co*-AMPS) elastomer** | No | 1.83 | 22 | 10.8 | 614 | S26 |
| | **Highly entangled elastomer** | No | 0.54 | ~14.5 | 2.2 | 240 | S20 |
| | **PDMS–MPU–IU elastomer** | Yes | 0.62 | ~14 | 12 | N/A | S27 |
| | **PEGA-co-MMA-Li elastomer** | Yes | 18 | 228 | ~95 | 2682 | S28 |
| **Ionogels** | **P(AAm-*co*-AA) ionogel** | Yes | 46.5 | ~44 | 24 | N/A | S29 |
| | **Microsphere-reinforced ionogel** | Yes | ~1 | 19 | 87 | 2120 | S30 |
| **Polyampholyte hydrogels** | **P(NaSS-*co*-DMAEA-Q) hydrogel 1** | Yes | 0.14 | ~9.75 | N/A | 71.1 | S31 |
| | **P(NaSS-*co*-DMAEA-Q) hydrogel 2** | Yes | ~0.8 | ~1.8 | ~3.65 | 150 | S32 |

Note: “N/A” indicates “not available” in the references.

**Supplementary Table 3.** Comparison of the stretchability, self-healability, and strain-stiffening properties among typical fiber mesh/fabric-reinforced hydrogels and elastomers.

| **Materials** | **Fiber** | | | **Composite** | | | |
|---|---|---|---|---|---|---|---|
| | **Fiber type** | **Stretchability** | **Self-healing** | **Stretchability** | **Self-healing** | **Strain-stiffening** | **Refs.** |
| **Hybrid ionic skin** | PU nanomesh | 1023% | Yes | 680% | Yes | Yes | This work |
| **Wood hydrogel** | Cellulose nanofiber | N/A | No | ~405% | No | Yes | S33 |
| **PCL scaffold-reinforced GelMA hydrogel** | 3D printed PCL scaffold | 50% | No | 43% | No | Yes | S34 |
| **PCL scaffold-reinforced IPN hydrogel** | 3D woven PCL scaffold | N/A | No | N/A | No | N/A | S35 |
| **Fiber-reinforced ECM** | Electrospun PCL fiber | N/A | No | N/A | No | N/A | S36 |
| **3D woven tissue scaffold** | 3D woven PGA yarn | N/A | No | 22-27% | No | N/A | S37 |
| **Silica nanofiber-reinforced alginate hydrogel** | Silica nanofiber network | N/A | No | N/A | No | N/A | S38 |
| **Silica nanofiber-reinforced PAAm hydrogel** | Silica nanofiber network | N/A | No | 1400% | No | No | S39 |
| **Hydrogel–mesh composite** | PET surgical mesh | N/A | No | ~40% | No | No | S40 |
| **Skeleton-matrix hydrogel** | 3D printed stiff hydrogel skeleton | ~275% | No | ~295% | No | No | S10 |
| **PE fiber/PAAm composite** | UHMWPE fiber | 6.3% | No | 150% | No | Yes | S41 |
| **Fabric-reinforced polyampholyte hydrogel** | Glass fabric | 2% | No | 4-8% | No | No | S42-S44 |
| **Fabric-reinforced viscoelastomer** | Carbon fiber fabric | 8.8% | No | 12.5% | No | No | S45 |
| **Anisotropic elastomer composite** | 3D polyimide skeleton | 80~295% | No | 80~150% | No | Yes | S46 |
| **Multi-nanofiber network-reinforced elastomer** | P(VDF-TrFE)/PU nanofiber | 120~160% | No | 125~160% | No | Yes | S47 |
| **Nanofiber-reinforced elastic conductor** | PVDF nanofiber | ~280% | No | ~350% | No | No | S48 |

Note: “N/A” indicates “not available” in the references.

**Supplementary Table 4.** A rough comparison of overall sensing-related performance between hybrid ionic skin and previously reported intrinsically stretchable ionic conductors.

| Materials | | Transmittance (%) | Strain-stiffening | Tensile strength (MPa) | Toughness (MJ $m^{-3}$) | Max. strain (%) | Max. gauge factor | Ionic conductivity (S $m^{-1}$) | Ref. |
|---|---|---|---|---|---|---|---|---|---|
| **Hybrid ionic skin** | | 80 | Yes | 14 | 24.4 | 680 | 66.8 | $2.0\times10^{-4}$ - 3.5 | This work |
| **P(AAm-co-AA)/HA/$CaCl_2$ ionic matrix** | | 96 | Yes | 1.0 | 6.7 | 1680 | 4 | $5.5\times10^{-4}$ - 4.8 | |
| **Hydrogels** | **PVA/HPC hydrogel** | Opaque | No | 1.3 | ~5.8 | 520 | 2 | 3.4 | S49 |
| | **PNA/PMA hydrogel fiber** | Yes | Yes | 2.3 | ~9.4 | 900 | ~1 | 0.69 | S50 |
| | **MAPAH fiber** | Opaque | Yes | 7 | 26.8 | 1200 | ~3.5 | 2 | S51 |
| | **SA/NaCl/PAM hydrogel** | 99.6 | Yes | 0.65 | 4.77 | 2000 | 2.7 | ~0.02 | S52 |
| **Ionogels** | **PU-IL ionogel** | Transparent | No | 1.54 | 2.5 | 327 | 1.5 | 0.12 | S53 |
| | **PMMA-r-PBA/IL gel** | 98.5 | No | 0.2 | ~1.6 | 850 | ~2.7 | 0.031 | S54 |
| | **PIL/IL gel** | 96.9 | No | ~0.8 | ~6.4 | 1400 | 4.7 | 0.02 | S55 |
| | **P(MEA-*co*-MTMA TFSI) ionogel** | 93 | Yes | 1.1 | ~7.5 | 1440 | 2 | 0.004 | S56 |
| **Eutectogels** | **Poly(PDES)-PA elastomer** | ~93 | No | ~0.425 | ~3.6 | 1300 | 2.1 | 0.078 | S57 |
| | **LA-based ionic conductor** | >85 | No | 0.152 | ~1.6 | 1092 | ~3.7 | 0.0028 | S58 |
| **Ionoelastomers** | **PBA/PIL ionoelastomer** | 96 | Yes | ~0.255 | ~1.2 | 1460 | 17.0 | 0.004 | S59 |
| | **P(MEA-*co*-IBA) ionic elastomer** | 90 | Yes | 1.5 | ~8 | 1640 | 6.0 | $1.59\times10^{-4}$ | S60 |
| | **PAA/betaine elastomer** | 99.7 | Yes | ~0.76 | ~6.2 | 1600 | 8.0 | $5.5\times10^{-4}$ – 0.04 | S61 |
| | **ACMO/TTEGDA ionic elastomer** | 92 | No | 0.2 | ~0.2 | 300 | 2 | $4.7\times10^{-4}$ | S62 |
| **Organohydrogels** | **PHEA/SA/Gly fiber** | 90 | No | 0.2 | ~0.4 | ~400 | ~1.9 | 0.765 | S63 |
| | **P(MEA-AA)/CMC/DMSO** | 100 | No | ~0.06 | ~0.24 | ~700 | ~3.6 | N/A | S64 |
| | **PVA/PAMAA/Gly/NaCl gel** | Opaque | No | 0.345 | 2.3 | 1002 | 8.3 | 1.4 | S65 |
| | **P(AAm-*co*-AA)/$FeCl_3$/Gly** | Transparent | No | 1.18 | ~3.2 | 500 | ~2.5 | $4.2\times10^{-3}$ | S66 |
| **Fiber-reinforced ionic conductors** | **CNF/PVA organohydrogel** | 87 | No | 1.4 | 5.25 | 660 | 1.5 | 3.2 | S67 |
| | **HAP/PVA organohydrogel** | ~60 | No | 0.36 | ~0.94 | 480 | 2.8 | 1 | S68 |
| | **Silica nanofiber/PAAm hydrogel** | 83 | No | 0.3 | 2.98 | 1400 | 2.7 | 3.93 | S39 |
| | **CMF/PDES elastomer** | 52 | No | 0.46 | 13.17 | 3210 | 3.7 | 0.09 | S69 |

Note: “N/A” indicates “not available” in the references.

**Supplementary Table 5.** Final results of the multiplication of the signs of each cross-peak in 2DCOS synchronous and asynchronous spectra of ionic matrix.

| | | | | | | | | |
|---|---|---|---|---|---|---|---|---|
| **1547** | - | - | - | - | - | - | - | |
| **1574** | + | + | - | - | - | - | | |
| **1603** | + | + | + | - | + | | | |
| **1622** | + | + | + | - | | | | |
| **1655** | + | + | + | | | | | |
| **1674** | + | + | | | | | | |
| **1687** | - | | | | | | | |
| **1714** | | | | | | | | |
| | **1714** | **1687** | **1674** | **1655** | **1622** | **1603** | **1574** | **1547** |

According to Noda's rule, the final specific order for ionic matrix during the water-absorbing process is given as follows: 1547 → 1687 → 1714 → 1574 → 1674 → 1622 → 1603 → 1655 $cm^{-1}$ (→ means prior to or earlier than), i.e. *v*($COO^-$) ($Ca^{2+}$ complexed) → *v*(C=O) (free PAAm) → *v*(COOH) (PAA) → *v*($COO^-$) (free) → *v*(C=O) (weakly H-bonded PAAm) → *δ*(O-H) ($H_2O$) → Amide II → *v*(C=O) (strongly H-bonded PAAm).

**Supplementary References**


S1. Noda, I. Two-dimensional infrared spectroscopy. *J. Am. Chem. Soc.* **111**, 8116-8118 (1989).

S2. Noda, I. Two-Dimensional Infrared (2D IR) spectroscopy: Theory and Applications. *Appl. Spectrosc.* **44**, 550-561 (1990).

S3. Sun, S. & Wu, P. Spectral insights into microdynamics of thermoresponsive polymers from the perspective of two-dimensional correlation spectroscopy. *Chin. J. Polym. Sci.* **35**, 700-712 (2017).

S4. Delalleau, A., Josse, G., Lagarde, J. M., Zahouani, H. & Bergheau, J. M. A nonlinear elastic behavior to identify the mechanical parameters of human skin in vivo. *Skin Res. Technol.* **14**, 152-164 (2008).

S5. Vatankhah-Varnosfaderani, M. et al. Chameleon-like elastomers with molecularly encoded strain-adaptive stiffening and coloration. *Science* **359**, 1509 (2018).

S6. Annaidh, A. N., Bruyère, K., Destrade, M., Gilchrist, M. D. & Otténio, M. Characterization of the anisotropic mechanical properties of excised human skin. *J. Mech. Behav. Biomed.* **5**, 139-148 (2012).

S7. Jansen, L. H. & Rottier, P. B. Some mechanical properties of human abdominal skin measured on excised strips. *Dermatology* **117**, 65-83 (1958).

S8. Pereira, B. P., Lucas, P. W. & Swee-Hin, T. Ranking the fracture toughness of thin mammalian soft tissues using the scissors cutting test. *J. Biomech.* **30**, 91-94 (1997).

S9. Lin, S., Liu, J., Liu, X. & Zhao, X. Muscle-like fatigue-resistant hydrogels by mechanical training. *Proc. Natl. Acad. Sci. U.S.A.* **116**, 10244-10249 (2019).
S10. Yang, H. et al. Fabricating hydrogels to mimic biological tissues of complex shapes and high fatigue resistance. *Matter* **4**, 1935-1946 (2021).
S11. Xiang, C., Wang, Z., Yang, C., Yao, X., Wang, Y. & Suo, Z. Stretchable and fatigue-resistant materials. *Mater. Today* **34**, 7-16 (2020).
S12. Liu, X. et al. Topoarchitected polymer networks expand the space of material properties. *Nat. Commun.* **13**, 1622 (2022).
S13. Liang, X. et al. Anisotropically fatigue-resistant hydrogels. *Adv. Mater.* **33**, 2102011 (2021).
S14. Lin, S. et al. Anti-fatigue-fracture hydrogels. *Sci. Adv.* **5**, eaau8528 (2019).
S15. Hua, M. et al. Strong tough hydrogels via the synergy of freeze-casting and salting out. *Nature* **590**, 594-599 (2021).
S16. Liang, X. et al. Bioinspired 2D isotropically fatigue-resistant hydrogels. *Adv. Mater.* **33**, 2107106 (2021).
S17. Lei, Z., Gao, W., Zhu, W. & Wu, P. Anti-fatigue and highly conductive thermocells for continuous electricity generation. *Adv. Funct. Mater.* **32**, 2201021 (2022).
S18. Zhang, W. et al. Fracture toughness and fatigue threshold of tough hydrogels. *ACS Macro Lett.* **8**, 17-23 (2019).
S19. Zhang, W. et al. Fatigue of double-network hydrogels. *Eng. Fract. Mech.* **187**, 74-93 (2018).
S20. Kim, J., Zhang, G., Shi, M. & Suo, Z. Fracture, fatigue, and friction of polymers in which entanglements greatly outnumber cross-links. *Science* **374**, 212-216 (2021).
S21. Lei, H. et al. Stretchable hydrogels with low hysteresis and anti-fatigue fracture based on polyprotein cross-linkers. *Nat.Commun.* **11**, 4032 (2020).
S22. Zhang, E., Bai, R., Morelle, X. P. & Suo, Z. Fatigue fracture of nearly elastic hydrogels. *Soft Matter* **14**, 3563-3571 (2018).
S23. Liu, C. et al. Tough hydrogels with rapid self-reinforcement. *Science* **372**, 1078 (2021).
S24. Li, C., Yang, H., Suo, Z. & Tang, J. Fatigue-Resistant elastomers. *J. Mech. Phys. Solids* **134**, 103751 (2020).
S25. Wang, Z., Xiang, C., Yao, X., Floch, P. L., Mendez, J. & Suo, Z. Stretchable materials of high toughness and low hysteresis. *Proc. Natl. Acad. Sci. U.S.A.* **116**, 5967 (2019).
S26. Zheng, Y. et al. Nanophase separation in immiscible double network elastomers induces synergetic strengthening, toughening, and fatigue resistance. *Chem. Mater*. **33**, 3321 (2021).
S27. Kang, J. et al. Tough and water-insensitive self-healing elastomer for robust electronic skin. *Adv. Mater.* **30**, 1706846 (2018).
S28. Li, M. et al. Superstretchable, yet stiff, fatigue-resistant ligament-like elastomers. *Nat. Commun.* **13**, 2279 (2022).
S29. Wang, M. et al. Tough and stretchable ionogels by in situ phase separation. *Nat. Mater.* **21**, 359 (2022).
S30. Li, W. et al. Recyclable, healable, and tough ionogels insensitive to crack propagation. *Adv. Mater.* **34**, 2203049 (2022).

S31. Li, X. et al. Mesoscale bicontinuous networks in self-healing hydrogels delay fatigue fracture. *Proc. Natl. Acad. Sci. U.S.A.* **117**, 7606 (2020).

S32. Li, X. et al. Effect of mesoscale phase contrast on fatigue-delaying behavior of self-healing hydrogels. *Sci. Adv.* **7**, eabe8210 (2021).

S33. Kong, W. et al. Muscle-inspired highly anisotropic, strong, ion-conductive hydrogels. *Adv. Mater.* **30**, 1801934 (2018).

S34. Visser, J. et al. Reinforcement of hydrogels using three-dimensionally printed microfibres. *Nat. Commun.* **6**, 6933 (2015).

S35. Liao, IC., Moutos, F. T., Estes, B. T., Zhao, X. & Guilak, F. Composite three-dimensional woven scaffolds with interpenetrating network hydrogels to create functional synthetic articular cartilage. *Adv. Funct. Mater.* **23**, 5833-5839 (2013).

S36. Xu, R., Taskin, M. B., Rubert, M., Seliktar, D., Besenbacher, F. & Chen, M. hiPS-MSCs differentiation towards fibroblasts on a 3D ECM mimicking scaffold. *Sci. Rep.* **5**, 8480 (2015).

S37. Moutos, F. T., Freed, L. E. & Guilak, F. A biomimetic three-dimensional woven composite scaffold for functional tissue engineering of cartilage. *Nat. Mater.* **6**, 162-167 (2007).

S38. Si, Y., Wang, L., Wang, X., Tang, N., Yu, J. & Ding, B. Ultrahigh-water-content, superelastic, and shape-memory nanofiber-assembled hydrogels exhibiting pressure-responsive conductivity. *Adv. Mater.* **29**, 1700339 (2017).

S39. Lu, X., Si, Y., Zhang, S., Yu, J. & Ding, B. In situ synthesis of mechanically robust, transparent nanofiber-reinforced hydrogels for highly sensitive multiple sensing. *Adv. Funct. Mater.* **31**, 2103117 (2021).

S40. Gao, Y. et al. Hydrogel-mesh composite for wound closure. *Proc. Natl. Acad. Sci. U.S.A.* **118**, e2103457118 (2021).

S41. Yi, F., Guo, F., Li, Y., Wang, D., Huang, P. & Fu, S. Polyacrylamide hydrogel composite E-skin fully mimicking human skin. *ACS Appl. Mater. Interfaces* **13**, 32084-32093 (2021).

S42. King, D. R. et al. Extremely tough composites from fabric reinforced polyampholyte hydrogels. *Mater. Horiz.* **2**, 584-591 (2015).

S43. Huang, Y. et al. Energy-dissipative matrices enable synergistic toughening in fiber reinforced soft composites. *Adv. Funct. Mater.* **27**, 1605350 (2017).

S44. Huang, Y. et al. Superior fracture resistance of fiber reinforced polyampholyte hydrogels achieved by extraordinarily large energy-dissipative process zones. *J. Mater. Chem. A* **7**, 13431-13440 (2019).

S45. Cui, W. et al. Fiber-reinforced viscoelastomers show extraordinary crack resistance that exceeds metals. *Adv. Mater.* **32**, 1907180 (2020).

S46. Ling, Y. et al. Bioinspired elastomer composites with programmed mechanical and electrical anisotropies. *Nat. Commun.* **13**, 524 (2022).

S47. Hanif, A. et al. A skin-inspired substrate with spaghetti-like multi-nanofiber network of stiff and elastic components for stretchable electronics. *Adv. Funct. Mater.* **30**, 2003540 (2020).

S48. Jin, H. et al. Highly durable nanofiber-reinforced elastic conductors for skin-tight electronic textiles. *ACS Nano* **13**, 7905-7912 (2019).

S49. Zhou, Y. et al. Highly stretchable, elastic, and ionic conductive hydrogel for artificial soft electronics. *Adv. Funct. Mater.* **29**, 1806220 (2019).
S50. Shuai, L., Guo, Z. H., Zhang, P., Wan, J., Pu, X. & Wang, Z. L. Stretchable, self-healing, conductive hydrogel fibers for strain sensing and triboelectric energy-harvesting smart textiles. *Nano Energy* **78**, 105389 (2020).
S51. Zhao, X., Chen, F., Li, Y., Lu, H., Zhang, N. & Ma, M. Bioinspired ultra-stretchable and anti-freezing conductive hydrogel fibers with ordered and reversible polymer chain alignment. *Nat. Commun.* **9**, 3579 (2018).
S52. Zhang, X. et al. Supramolecular nanofibrillar hydrogels as highly stretchable, elastic and sensitive ionic sensors. *Mater. Horiz.* **6**, 326-333 (2019).
S53. Li, T., Wang, Y., Li, S., Liu, X. & Sun, J. Mechanically robust, elastic, and healable ionogels for highly sensitive ultra-durable ionic skins. *Adv. Mater.* **32**, 2002706 (2020).
S54. Kim, Y. M. & Moon, H. C. Ionoskins: nonvolatile, highly transparent, ultrastretchable ionic sensory platforms for wearable electronics. *Adv. Funct. Mater.* **30**, 1907290 (2020).
S55. Yu, Z. & Wu, P. Underwater communication and optical camouflage ionogels. *Adv. Mater.* **33**, 2008479 (2021).
S56. He, C., Sun, S. & Wu, P. Intrinsically stretchable sheath-core ionic sensory fibers with well-regulated conformal and reprogrammable buckling. *Mater. Horiz.* **8**, 2088-2096 (2021).
S57. Li, Ra., Fan, T., Chen, G., Xi, H., Su, B. & He, M. Highly transparent, self-healing conductive elastomers enabled by synergistic hydrogen bonding interactions. *Chem. Eng. J.* **393**, 124685 (2020).
S58. Dang, C. et al. Transparent, highly stretchable, rehealable, sensing, and fully recyclable ionic conductors fabricated by one-step polymerization based on a small biological molecule. *Adv. Funct. Mater.* **29**, 1902467 (2019).
S59. Ming, X., Zhang, C., Cai, J., Zhu, H., Zhang, Q. & Zhu, S. Highly transparent, sretchable, and conducting ionoelastomers based on Poly(ionic liquid)s. *ACS Appl. Mater. Interfaces* **13**, 31102-31110 (2021).
S60. Yiming, B. et al. A mechanically robust and versatile liquid-free ionic conductive elastomer. *Adv. Mater.* **33**, 2006111 (2021).
S61. Zhang, W., Wu, B., Sun, S. & Wu, P. Skin-like mechanoresponsive self-healing ionic elastomer from supramolecular zwitterionic network. *Nat. Commun.* **12**, 4082 (2021).
S62. Wang, L., Wang, Y., Yang, S., Tao, X., Zi, Y. & Daoud, W. A. Solvent-free adhesive ionic elastomer for multifunctional stretchable electronics. *Nano Energy* **91**, 106611 (2022).
S63. Song, J. et al. Mechanically and electronically robust transparent organohydrogel fibers. *Adv. Mater.* **32**, 1906994 (2020).
S64. Liu, J. et al. Ionic conductive organohydrogels with dynamic pattern behavior and multi-environmental stability. *Adv. Funct. Mater.* **31**, 2101464 (2021).
S65. Huang, J. et al. Self-powered integrated system of a strain sensor and flexible all-solid-state supercapacitor by using a high performance ionic organohydrogel. *Mater. Horiz.* **7**, 2085-2096 (2020).
S66. Ju, M., Wu, B., Sun, S. & Wu, P. Redox-active iron-citrate complex regulated robust

coating-free hydrogel microfiber net with high environmental tolerance and sensitivity. *Adv. Funct. Mater.* **30**, 1910387 (2020).

S67. Ye, Y., Zhang, Y., Chen, Y., Han, X. & Jiang, F. Cellulose nanofibrils enhanced, strong, stretchable, freezing-tolerant ionic conductive organohydrogel for multi-functional sensors. *Adv. Funct. Mater.* **30**, 2003430 (2020).

S68. Wen, J. et al. Multifunctional ionic skin with sensing, UV-filtering, water-retaining, and anti-freezing capabilities. *Adv. Funct. Mater.* **31**, 2011176 (2021).

S69. Sun, X., Zhu, Y., Zhu, J., Le, K., Servati, P. & Jiang, F. Tough and ultrastretchable liquid-free ion conductor strengthened by deep eutectic solvent hydrolyzed cellulose microfibers. *Adv. Funct. Mater.* **32**, 2202533 (2022).
