# Supplementary material for: Fatigue-free artificial ionic skin toughened by self-healable elastic nanomesh
Source: Nat Commun. 2022 Jul 29;13:4411. doi: 10.1038/s41467-022-32140-3 (PMC9338060; doi:10.1038/s41467-022-32140-3)
Supplement: Supplementary file 2 — Description of Additional Supplementary Information [file 41467_2022_32140_MOESM2_ESM.docx]

**Description of Additional Supplementary Files**

File name: Supplementary Movie 1

Description: Strain-stiffening demonstration of hybrid ionic skin

File name: Supplementary Movie 2

Description: Single-edge notch tension test of hybrid ionic skin

File name: Supplementary Movie 3

Description: Self-healing of hybrid ionic skin
